# Interpractice variability in antibiotic prescribing for acute respiratory tract infections: a cross-sectional study of Australian early-career general practitioners

**DOI:** 10.1136/bmjopen-2024-094811

**Published:** 2025-08-03

**Authors:** Alexandria Turner, Mieke L van Driel, Ben Mitchell, Elizabeth Holliday, Josh Davis, Amanda Tapley, Andrew Davey, Anna Ralston, Jason Dizon, Emma Baillie, Alison Fielding, Katie Mulquiney, Lisa Clarke, Neil Spike, Parker Magin

**Affiliations:** 1 General Practice Clinical Unit, Faculty of Medicine, The University of Queensland, Brisbane, Queensland, Australia; 2 GP Training Research, Royal Australian College of General Practitioners, Mayfield West, New South Wales, Australia; 3 School of Medicine and Public Health, The University of Newcastle, Callaghan, New South Wales, Australia; 4 Data Sciences, Hunter Medical Research Institute, New Lambton Heights, New South Wales, Australia; 5 GP Training Medical Education, Royal Australian College of General Practitioners, Hobart, New South Wales, Australia; 6 Department of General Practice and Primary Health Care, The University of Melbourne, Carlton, Victoria, Australia; 7 School of Rural Health, Monash University, Churchill, Victoria, Australia; 8 School of Public Health and Community Medicine, University of New South Wales, UNSW Medicine, Kensington, New South Wales, Australia

**Keywords:** Antibiotics, Respiratory infections, MEDICAL EDUCATION & TRAINING, Primary Care

## Abstract

**Abstract:**

**Objectives:**

Frequency of general practitioners’ (GPs’) antibiotic prescribing for acute, self-limiting respiratory tract infections (aRTIs) is high. The practice environment and culture influence the clinical behaviour, including prescribing behaviour, of GP specialist vocational trainees (registrars). We aimed to assess inter-practice variability in registrars’ antibiotic prescribing.

**Design:**

This was a cross-sectional analysis from the Registrar Clinical Encounters in Training (ReCEnT) cohort study, from 2010 to 2020.

**Setting:**

ReCEnT documents registrars’ clinical experiences and behaviours. Before 2016, 5 of 17 Australian training regions participated in ReCEnT. From 2016, three of nine regions (~40% of Australian registrars) participated.

**Participants:**

3210 registrars (response rate 91.8%) from 1286 training practices contributed to the analysis.

**Outcome measures:**

The outcomes were prescription of an antibiotic for new diagnoses of (1) all aRTIs and (2) acute bronchitis diagnoses specifically. Prescribing percentages were calculated at the training practice level. Intraclass correlation coefficients (ICCs) were used to measure the ratio of interpractice variation to total variance. Median ORs (MORs) were also estimated to quantify interpractice variability.

**Results:**

Practice-level antibiotic prescribing percentages ranged from 0% to 100% for both aRTIs and acute bronchitis diagnoses in the primary analysis. ICCs for aRTI prescribing were 0.08 (unadjusted) and 0.02 (adjusted). For acute bronchitis, ICCs were 0.10 (unadjusted) and 0.05 (adjusted). MORs were 1.66 (unadjusted) and 1.32 (adjusted) for aRTIs. MORs for acute bronchitis were 1.80 (unadjusted) and 1.53 (adjusted). This indicates a marked variation in the odds of a patient receiving antibiotics for an aRTI if randomly attending different practices.

**Conclusions:**

There was considerable interpractice variation in registrars’ antibiotic prescribing frequencies. Further research is required to examine the factors accounting for this variation and to develop practice-level interventions to reduce antibiotic prescribing in high-prescribing practices.

**STRENGTHS AND LIMITATIONS OF THIS STUDY**

- The sample frame reflects a large, geographically diverse representative sample of Australian general practice teaching practices.
- The analyses include detailed, contemporaneously recorded, in-consultation prescribing data linked to the problem/diagnoses.
- This study uses two measures of interpractice variability in antibiotic prescribing for respiratory infections in an Australian setting.
- Causality cannot be inferred using a cross-sectional design.
- Our data do not include a direct measure of illness severity or whether an antibiotic script was filled by the patient.

## Introduction

 Antibiotic resistance is a global health concern, with over one million attributable deaths in 2019.^1^ Antibiotic overprescribing is a major contributor to antibiotic resistance.^2^ Most antibiotic prescriptions are written in the primary care setting.^3^ Antibiotic overprescribing is prescribing for conditions for which antibiotics do not provide substantial benefit, including acute, self-limiting, respiratory infections.^4^

Australia ranks seventh highest in terms of community antibiotic use when compared with 30 European countries and Canada.^2^ In 2019, 40.3% of the Australian population was dispensed at least one systemic antibiotic.^2^ In Australia, prescribing frequencies are well above the recommended benchmarks for respiratory tract infections. For all acute respiratory infections, the ideal antibiotic prescribing percentage is benchmarked as under 20% of presentations.^4^,^6^ However, in Australia, 57% of all acute respiratory infections are managed with antibiotics.^4^ Despite a downward trend over the past decade, greater reductions in antibiotic prescribing frequencies for acute respiratory tract infections (aRTIs) are required to curb the growth of antimicrobial resistance.^2 7^

Research in the UK primary care setting has reported substantial variability between practice prescribing frequencies for respiratory tract infections.^8^,^10^ In a large study, antibiotics were prescribed for between 39% (lowest decile of practices) and 69% (highest decile of practices) of respiratory tract infections.^9^ Between-practice differences remain apparent when adjusted for other key explanatory factors including variation in patient comorbidities, immunosuppressant use and demographics, as well as general practitioner (GP) consultation rates.^11 12^ This suggests that practice culture contributes to the decision to prescribe antibiotics. However, a GP’s individual decision to prescribe antibiotics is complex. This decision must take into account multiple factors including, but not limited to evidence for effectiveness of antibiotics, patient expectations, clinical uncertainty regarding risk of complications, as well as practice culture.^13^

Antibiotic prescribing by GP registrars (vocational trainees in general practice) is particularly important. Approximately 13% of the Australian GP workforce by head-count in 2020–2021 were registrars.^14^ There is evidence that GPs’ prescribing habits, once established, are stable over time,^15^ although behaviour change can occur following educational interventions.^16 17^ Despite having lower prescribing frequencies than established GPs, Australian GP registrars’ antibiotic prescribing frequencies are still above recommended benchmarks.^4 7 18^ Qualitative research suggests the prescribing ‘culture’ of individual training practices influences registrars’ antibiotic prescribing (to a more parsimonious or more liberal pattern of prescribing).^16 19^

In this study, we aimed to assess variability between Australian training practices in GP registrars’ antibiotic prescribing following their diagnoses of any of a range of common acute, self-limiting, respiratory infections and for acute bronchitis, specifically. Acute bronchitis was selected as the exemplar acute, self-limiting, respiratory infections for which antibiotics are not indicated.

## Methods

### Study design

This was a cross-sectional analysis of data from the Registrar Clinical Encounters in Training (ReCEnT) cohort study, encompassing 22 rounds of 6 monthly data collection from 2010 to 2020.

### Registrar Clinical Encounters in Training

ReCEnT is an ongoing inception cohort study of GP registrars.^20 21^ General practice vocational training in Australia is an apprenticeship-like training system where registrars practise under the supervision of established GPs in the community. GP registrars have access to their supervisor but practise with considerable autonomy. This includes having equivalent prescribing rights to those of their more senior GP colleagues. GP registrars receive educational sessions from regional training organisations to complement their experiential in-practice learning.

Prior to 2016, 5 of Australia’s 17 regional training organisations participated in ReCEnT. Since 2016, subsequent to a restructure of Australian GP vocational training, three of Australia’s nine training organisations participate in ReCEnT (training ~40% of all Australian registrars).^22^

Within ReCEnT, registrars record their clinical experiences and behaviours, including their prescribing behaviour. Detailed ReCEnT methods have been documented previously.^20 23^ Briefly, registrars record demographic details of themselves and their training practice and then document 60 consecutive consultations during each of their three training terms. These data relate to patient and consultation factors including problems/diagnoses dealt with and any prescribing performed (specifically linked by registrars in their data recording to the relevant problems/diagnoses that prompted the prescription). Registrars describe each problem/diagnosis in their own words as accurately as possible. Registrars’ responses (for problems/diagnoses) are coded to the International Classification of Primary Care-2 PLUS (ICPC-2PLUS) system^24^ by trained coders.

Only office-based consultations are included in data collection. Home visits and visits to aged care facilities are not recorded. The demographic characteristics of registrars in participating regions are similar to those of Australian registrars in general.^22^

Participation in ReCEnT as an educational exercise is a required component of all registrars’ educational programme during each of their three training terms. Each registrar is provided with an individual report following each data-collection period. This allows for reflection on their experiences and learning.^21 25^ Registrars are also given the option to provide consent for their data to also be used for research purposes. These analyses only include data from registrars who provided consent to use their data for research purposes.

### Outcome

The outcome of interest was binary: prescription of a systemic antibiotic (defined as J01 in the Anatomical Therapeutic Chemical (ATC) classification system)^26^ versus a systemic antibiotic not being prescribed.

In the ReCEnT study, registrars’ recording of the names of medicines prescribed is coded to ATC codes (by trained coders as, above, for problems/diagnoses and ICPC-2PLUS). Thus, ATC-coded medicines are linked to the relevant ICPC-2PLUS problems/diagnoses for which they were prescribed.

### Independent variables

Explanatory variables included in analyses were measured at the practice, patient, registrar and consultation levels. The practice factors were: size of practice (‘small’ 1–4, ‘large’ > 4 full-time equivalent GPs), bulk billing status (whether the practice routinely requires no patient contribution to the cost of the consultation), rurality,^27^ Socio-Economic Indexes for Areas Index of Relative Social Disadvantage^28^ decile of the practice location, analysed as a continuous variable; and training region. The patient factors were age, gender, Aboriginal and Torres Strait Islander status, non-English speaking background status, and whether the patient was new to the registrar, new to the practice or a returning patient. The registrar-level factors were age, gender, part time/full-time status, training term, worked at practice before and source of primary medical degree (qualified as a doctor in Australia or internationally). The consultation factors were consultation duration and accessing of supervisor advice or assistance.

### Statistical analysis

#### Analysis population

Analyses were performed at the problem/diagnosis level and were restricted to the following problems/diagnoses, defined as ICPC-2PLUS codes:

- New, self-limiting aRTIs (acute upper respiratory tract infection, R74; acute bronchitis/bronchiolitis, R78; sinusitis acute/chronic, R75; streptococcal throat, R72; tonsillitis acute, R76; and acute otitis media/myringitis, H71; for one set of analyses (hereafter referred to as ‘aRTIs’).
- New, acute bronchitis problems (ICPC-2PLUS code: R78) for a second set of analyses (hereafter referred to as ‘bronchitis’).

The rationale for the two chosen analyses was that analysing all (commonly seen in general practice) aRTIs provides a summation of interpractice variability of the relevant prescribing. However, this encompasses infections with heterogeneity, including infections with different proportions of acceptable antibiotic prescribing according to Australian^4^ and international benchmarks.^5 6^ For some of the included infections, evidence-based guidelines provide criteria for prescribing an antibiotic (applicable to a modest proportion of cases). A separate analysis focused on bronchitis provides evidence for interpractice variability in an exemplar condition for which the applicable Australian evidence-based guidelines stipulate no antibiotic prescribing,^29^ but for which our previous research suggests high proportions of prescribing by registrars.^7 18 30^

#### Descriptive statistics

Descriptive statistics were reported as mean and SD or median and IQR, as appropriate, for continuous variables and as frequencies with proportions for categorical variables.

Practice prescribing proportions were estimated by dividing the number of times an antibiotic was prescribed for a diagnosis (of aRTI or bronchitis), by the total number of new diagnoses (of aRTI or bronchitis) seen by each practice.

#### Inferential statistical methods

For aRTI and bronchitis, two statistical measures were used to estimate between-practice variability: intraclass correlation coefficients (ICCs) and median ORs (MORs).^31 32^

Mixed-effects logistic regression models were used to examine the effects of explanatory variables on practice antibiotic prescribing frequency. These models included random effects to account for repeated measures on registrars and practices over time. The Laplace approximation was used for parameter estimation.

#### Intraclass correlations

ICCs were used to estimate the proportion of total outcome variation attributable to between-practice outcome variation.

When the model included only a random effect for practice (model 1), ICCs were estimated as:

$ICC=\frac{\sigma_{P}^{2}}{\sigma_{P}^{2}+3.29}$

When the model included random effects for both practice and registrar (models 2–6), ICCs were estimated as:

$ICC=\frac{\sigma_{P}^{2}}{\sigma_{P}^{2}+\sigma_{R}^{2}\;+3.29}$

Where:

- $\sigma_{P}^{2}$ represents between-practice variance.
- $\sigma_{R}^{2}$ represents between-registrar variance.
- $\frac{\pi^{2}}{3}=$3.29=logistic distribution variance, used to represent the level-1 residual variance.^33^

#### Median ORs

MORs were also estimated to quantify between-practice variation in antibiotic prescribing for new aRTI or bronchitis, controlling for covariates.

The MOR represents the median multiplicative increase in the odds of antibiotic prescribing between a practice with higher prescribing odds and a practice with lower prescribing odds, across all randomly sampled pairs of practices, adjusted for any covariates in the model. The MOR can be interpreted as the median increase in the odds of being prescribed an antibiotic for the diagnosis of interest if a patient moved from a lower risk to a higher risk practice. If there is no practice-level variation, the MOR will be 1.

MORs were estimated as follows:

$MOR=\exp\left(0.95\cdot \sqrt{\sigma_{P}^{2}}\right)$

#### Model construction

The first model included a random effect for practice. The second model included random effects for practice and registrar to account for correlation within practices due to repeated measures on registrars. ‘Initially, ICCs and MORs were estimated for each outcome to assess the proportion of variation in the outcome existing between practices, without and with accounting for repeated measures on registrars, without adjusting for any explanatory variables (these are called ‘empty models’)’. Four additional models (including the random effects for practice and registrar) were constructed by progressively including additional factor groups, to assess the relative change in between-practice prescribing variation controlling for the effects of covariates (see tables 3 and 4 for model details).

Missing data were handled using complete case analyses. No imputation was performed to address missing data (online supplemental tables 1–4).

#### Sensitivity analysis

In the primary analyses, all training practices were included. Sensitivity analyses included only practices where three or more registrars contributed data. This sensitivity analysis was performed to evaluate the robustness of results from primary analysis to reduce potential bias by the inclusion of practices contributing smaller numbers of respiratory infections.

All analyses were programmed using STATA V.16.0 and SAS V.9.4.

### Patient and public involvement

Patients and/or the public were not involved in the design, or conduct, or reporting, or dissemination plans of our research. The participants in this study are GP registrars. GP registrars were involved in the original design of the ReCEnT study in 2009.

## Results

The response rate for registrars during the 2010–2020 study period was 91.8%. 3210 registrars (response rate 91.8%) from 1286 training practices contributed to the analysis.

In the primary analysis, there were 3175 registrars from 1278 practices that contributed to 50 589 aRTI diagnoses. Of these, 18 309 (36.2%) had a systemic antibiotic prescribed. The registrars were mostly female (61.5%) and completed their primary medical degree in Australia (81.0%). The average registrar age was 32.6 years. Practices were primarily larger (61.4%), major city (62.3%) practices (table 1).

**Table 1:** Registrar and registrar-round/practice characteristics

Variables	Class	Acute self-limiting respiratory infections	Acute bronchitis
		Total, n (%)	
Registrar characteristics		(N=3175)	(N=2059)
Registrar gender	Female	1952 (61.5)	1281 (62.2)
Country of primary medical degree	Australia	2520 (81.0)	1613 (79.7)
Years of previous medical work	Mean±SD	3.4 (3.3)	3.4 (3.3)
Pathway registrar enrolled in	Rural	954 (30.5)	614 (30.1)
Has post-graduate qualifications	Yes	1025 (32.9)	678 (33.4)
College seeking fellowship	RACGP	2950 (92.9)	1932 (93.8)
	ACCRM	71 (2.2)	30 (1.5)
	Both	30 (0.9)	20 (1.0)
Year of graduation	Mean±SD	2010.0 (5.7)	2009.6 (5.8)
Registrar-round/practice characteristics		(N=7686)	(N=3258)
Registrar age (years)	Mean (SD)	32.6 (6.2)	32.6 (6.3)
Registrar works full-time	Yes	5635 (77.1)	2455 (79.0)
Registrar training term	Term 1	2953 (38.4)	1176 (36.1)
	Term 2	2707 (35.2)	1207 (37.1)
	Term 3	2026 (26.4)	875 (26.9)
Registrar does other medical work	Yes	1304 (18.2)	559 (18.4)
Registrar worked at practice previously	Yes	1709 (23.6)	747 (24.2)
Size of practice	Large (6+FTE GPs)	4488 (61.4)	1977 (63.5)
Practice routinely bulk bills	Yes	2223 (30.3)	857 (27.4)
Rurality of practice	Major city	4785 (62.3)	2013 (61.8)
	Inner regional	2073 (27.0)	895 (27.5)
	Outer regional/remote/very remote	827 (10.8)	350 (10.7)
SEIFA decile of practice	Mean (SD)	5.54 (2.8)	5.7 (2.8)

ACCRM, Australian College of Rural and Remote Medicine; FTE, full-time equivalent; GP, general practitioner; RACGP, Royal Australian College of General Practitioners; SEIFA, Socio-Economic Indexes For Areas.

Within the bronchitis diagnoses study population, there were 2059 registrars from 992 practices that contributed to a total of 5552 bronchitis diagnoses. Of these, 4111 (74.1%) had a systemic antibiotic prescribed. Registrar and practice characteristics were similar across both study populations (table 1).

The sensitivity analyses, excluding practices with <4 registrars, included 2963 registrars across 692 practices contributing to 43 409 aRTI consultations (36.5% of which a systemic antibiotic was prescribed) and 1840 registrars from 664 practices contributing to 4821 bronchitis consultations (74.8% of which a systemic antibiotic was prescribed).

The mean prescribing percentage across all practices was 36% (95% CI 35% to 37%) for aRTIs. For bronchitis, the mean practice prescribing percentage was 72% (95% CI 70% to 74%) (table 2).

**Table 2:** Practice antibiotic prescribing rates for acute, self-limiting respiratory infections and acute bronchitis problems

	Acute self-limiting respiratory infections		Acute bronchitis	
	Mean (95% CI)	Median (IQR)	Mean (95% CI)	Median (IQR)
Primary analysis	36% (35% to 37%)	34% (24%–46%)	72% (70% to 74%)	80% (50%–100%)
Sensitivity analysis	36% (35% to 37%)	36% (27%–44%)	74% (71% to 76%)	79% (60%–100%)

**Figure 1:**
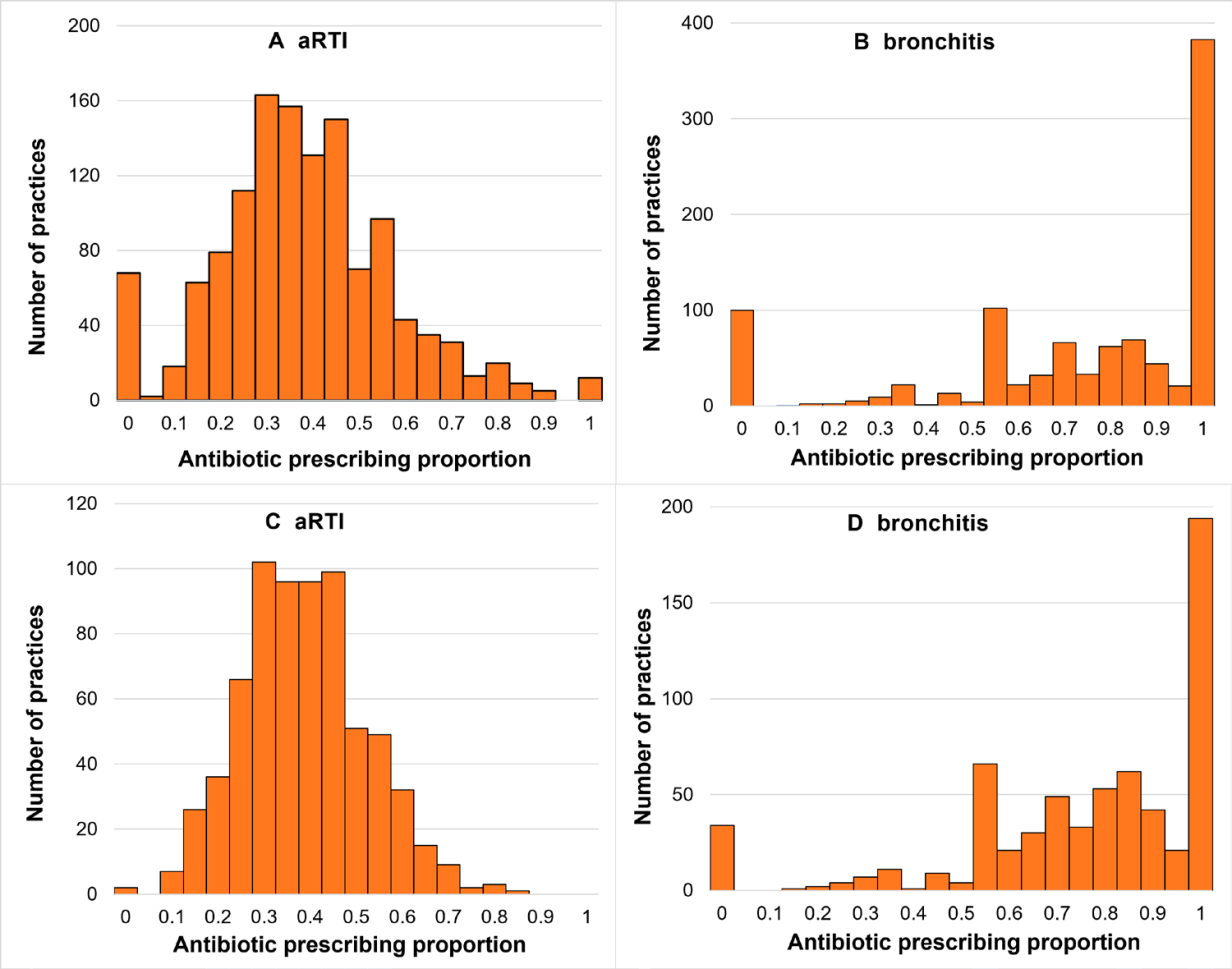
Distribution of practice-specific antibiotic prescribing proportions for acute self-limiting upper respiratory infections (**A**), acute bronchitis (**B**), acute self-limiting upper respiratory infections (sensitivity analysis); (**C**) and acute bronchitis (sensitivity analysis); (**D**). aRTI, acute respiratory tract infection.

Practice-specific prescribing percentages were between 0% and 100% for both aRTI (n=1278; figure 1A) and bronchitis (n=992; figure 1B) diagnoses. In the sensitivity analysis, practice prescribing percentages for all aRTI ranged from 0% to 86% (n=692; figure 1C) and from 0% to 100% for bronchitis (n=644; figure 1D).

For diagnoses of aRTIs, the ICC was 0.08 and the MOR was 1.66 in the unadjusted model (model 1), including a random effect for practice. In the final model (model 6), adjusted for multiple individual and cluster characteristics, with random effects for practice and registrar, the ICC decreased to 0.02 and the MOR to 1.32 (see table 3 for all models). The results from the sensitivity analyses were not substantively different.

**Table 3:** Antibiotic prescribed for new diagnosis of acute, self-limiting respiratory tract infection (upper respiratory tract infection, acute sore throat, acute bronchitis, acute otitis media, acute sinusitis)

Model		Primary analysis (n=50 589)		Sensitivity analysis (n=43 409)	
		ICC	MOR	ICC	MOR
Model 1	Empty model, with a random effect for practice	0.079	1.655	0.062	1.558
Model 2	Empty model, with random effects for practice and registrar	0.033	1.410	0.033	1.405
Model 3	Adjusted for registrar factors, including random effects for practice and registrar	0.030	1.378	0.028	1.368
Model 4	Adjusted for registrar and patient factors, including random effects for practice and registrar	0.027	1.355	0.024	1.338
Model 5	Adjusted for registrar, patient and consultation factors, including random effects for practice and registrar	0.029	1.374	0.027	1.356
Model 6	Adjusted for registrar, patient, consultation and practice factors, including random effects for practice and registrar	0.023	1.323	0.020	1.301

ICC, intraclass correlation coefficient; MOR, median OR.

When considering new diagnoses of bronchitis, the ICC was 0.10 and the MOR was 1.80 in the unadjusted model (model 1), including only a random effect for practice. In the final adjusted model (model 6) with random effects for practice and registrar, the ICC decreased to 0.05 and the MOR decreased to 1.53 (see table 4 for all models). The results from the sensitivity analyses were not substantively different.

**Table 4:** Antibiotic prescribed for new diagnosis of acute bronchitis

Model		Primary analysis (n=5552)		Sensitivity analysis (n=4821)	
		ICC	MOR	ICC	MOR
Model 1	Empty model, with a random effect for practice	0.103	1.795	0.087	1.703
Model 2	Empty model, with random effects for practice and registrar	0.044	1.512	0.043	1.505
Model 3	Adjusted for registrar factors, including random effects for practice and registrar	0.050	1.553	0.048	1.540
Model 4	Adjusted for registrar and patient factors, including random effects for practice and registrar	0.053	1.588	0.049	1.561
Model 5	Adjusted for registrar, patient and consultation factors, including random effects for practice and registrar	0.053	1.589	0.048	1.558
Model 6	Adjusted for registrar, patient, consultation and practice factors, including random effects for practice and registrar	0.046	1.533	0.041	1.498

ICC, intraclass correlation coefficient; MOR, median OR.

See online supplemental table 5 for missingness of data in all models, and online supplemental tables 6–9 for primary and sensitivity regression models for aRTI and bronchitis. See online supplemental table 10 for proportion of variability attributable to practice and to registrar in each of the models.

## Discussion

### Statement of principal findings

The mean prescribing percentage across all practices was 36% for aRTIs and 72% for bronchitis. Practice antibiotic prescribing percentages ranged from 0% to 100% for both aRTIs and bronchitis in the primary analysis. The distribution of all practices prescribing percentages for aRTIs showed a spike at 0% but was otherwise approximately normal. Comparison with the distribution for the sensitivity analysis for aRTIs suggested the spike at 0% was attributable to practices with a small sample size of registrars and their consultations. For bronchitis presentations, however, there were prominent spikes at 0% and 100% and, while attenuated, these were still prominent in the sensitivity analysis.

We found evidence in our ICCs, for both aRTIs and bronchitis, including with adjustment for multiple potential confounders, of non-negligible correlation (see below, ‘Interpretation within the context of the wider literature’, for a discussion of ranges of ICC values).

Our MOR findings indicate marked variation in the odds of a patient receiving antibiotics for a respiratory infection (adjusted MOR 1.32), particularly for bronchitis (adjusted MOR 1.53), when moving randomly between practices. The ICCs and MOR values were similar across the primary and sensitivity analyses.

### Strengths and limitations

The ReCEnT study has a very high response rate for studies of GPs,^34^ a large sample size, representative coverage of urban and rural practices, and a direct linkage of prescription and diagnosis for which the prescription was made.

A key strength of this study was the use of two measures of variability (ICC and MOR) between practice prescribing odds. Importantly, the calculation of the MORs in this study allowed for easily interpretable quantification of the level of variability between practices.

The adjustment for a large number of patient and practice factors previously associated with higher antibiotic prescribing^10^ is a strength.

In terms of limitations, the ReCEnT study does not have a measure of illness severity. However, in Australian peak evidence-based clinical guidelines, symptom severity is not a major determining factor for prescribing for aRTIs (and no prescribing is recommended in bronchitis irrespective of severity).^29^

Another consideration is the number of bronchitis presentations compared with aRTIs. While the total number of bronchitis presentations is considerable, some practices may have had low numbers of bronchitis presentations, potentially increasing interpractice variability. The consistency of primary and sensitivity analyses, however, argues against this.

ReCEnT does not contain information about whether the prescription was filled; however, the focus of this study is GPs’ prescribing behaviour. Additionally, our analyses cover a 10-year period. It is possible there were changes in prescribing habits over time that varied between practices; however, we anticipate such changes would have negligible impact on measures of interpractice variability.

In terms of generalisability, the results are strongly generalisable to Australian general practice training but may not be so to training programmes in other countries, especially those countries that (unlike Australia, the UK, Ireland, New Zealand and a number of other countries) do not have apprenticeship-like training models, or that have different prescribing regulatory regimens.

### Interpretation within the context of the wider literature

Our findings of considerable interpractice variability are consistent with ranges of practice antibiotic prescribing in a large UK study: 0%–97%, 7%–100% and 0%–75% for adult sore throat, adult acute lower respiratory tract infection, and children’s acute cough/respiratory infection cohorts, respectively.^8^

#### Intraclass correlation coefficients

ICCs for antibiotic prescribing for aRTIs ranged from 0.08 (unadjusted) to 0.02 (adjusted) and, for bronchitis, ranged from 0.10 (unadjusted) to 0.05 (adjusted). While these ICC values may appear low (ICC of 1 equals perfect correlation and 0 equals no correlation), these figures are similar to (and in some cases greater than) other ICCs calculated for primary care practice outcomes. A previous study calculated ICCs for 17 primary care clinical trials.^35^ The unadjusted median ICC was 0.016, and 0.011 when adjusted for baseline covariates.^35^ Another UK study that examined variation between practices for a range of general practice outcomes reported a median ICC of 0.051 (IQR 0.011–0.094).^36^ A further study which more broadly analysed data from 31 cluster-based studies in primary care reported that adjustments for individual or cluster-level characteristics reduced the magnitudes of the ICCs.^37^ This study reported an unadjusted median ICC (IQR) of 0.001 (0–0.0320) and an adjusted median ICC (IQR) of 0.005 (0–0.021) after adjusting for individual and cluster level variables.^37^

It is important to note that these studies include a broad range of outcomes; however, they give some context for ICC magnitudes in the general practice setting. While appearing low, our ICC values were considerably higher than those reported in these previous studies, suggesting a high level of clustering at the practice level for antibiotic prescribing for respiratory infections and bronchitis.

#### Median ORs

MOR values for antibiotic prescribing for aRTIs ranged from 1.65 (unadjusted) to 1.32 (adjusted), and for bronchitis, ranged from 1.80 (unadjusted) to 1.53 (adjusted) in the primary analysis. A recent UK study that also described variations in practice prescribing percentages reported MORs of 2.5 for sore throat, 2.9 for adult cough data and 2.1 for children’s cough data.^8^ Some key differences in this study include use of GP rather than registrar data, different categories of respiratory infections and adjustment for a different range of variables. There are differences between Australian and UK general practice at the wider level, but one potential explanation of the lower MORs in our study may be that registrars, by definition, work in teaching practices rather than unselected practices in the UK study. It is reasonable to assume that these are ‘higher compliance/quality’ practices than non-teaching practices, with greater adherence to evidence-based practice guidelines.^38^ The implication being that our MORs may be underestimates of inter-practice variability in the wider GP practice population.

### Implications for policy, practice and research

The registrars’ high prescribing percentage for bronchitis may relate to clinical uncertainty (particularly around the differential diagnosis of pneumonia), patient demand, clinician misconceptions and lack of accountability for prescribing.^39^ Qualitative evidence suggests registrar prescribing for bronchitis is also much influenced by the prescribing habits of their supervisors and other GPs in the practice.^16 19^

As registrars’ antibiotic prescribing has previously been shown to be amendable to educational intervention delivered to both registrars and their supervisors,^17^ practice ‘culture’ should, thus, also be a target for interventions to reduce registrars’ prescribing.

Potential strategies for practice-level approaches to improve antibiotic prescribing for respiratory infections may include: whole-practice protocols for common respiratory infections, regular clinical meetings with an antibiotic stewardship focus, practice waiting-room posters to engage patients, evidence-based leadership by senior GPs or training supervisors and implementation of audit and feedback processes.^40^ Similar system-level interventions have previously been shown to be effective in improving antibiotic prescribing in hospital settings.^41^

## Conclusions

Our findings indicate considerable between-practice variation in the odds of a patient receiving antibiotics for a respiratory infection, particularly for bronchitis. This suggests practice environment or culture may influence registrars’ prescribing habits and that future practice-level interventions regarding antibiotic stewardship are indicated to improve registrars’ prescribing practice.

## Supplementary material

10.1136/bmjopen-2024-094811 online supplemental file 1

## Data Availability

No data are available.
