# Supplementary material for: Interpractice variability in antibiotic prescribing for acute respiratory tract infections: a cross-sectional study of Australian early-career general practitioners
Source: BMJ Open. 2025 Aug 3;15(8):e094811. doi: 10.1136/bmjopen-2024-094811 (PMC12320071; doi:10.1136/bmjopen-2024-094811)
Supplement: online supplemental file 1 [file bmjopen-15-8-s001.docx]

### Supplementary Table 1. Missing data details from the primary analysis of acute self-limiting respiratory infection diagnoses.

| **Group** | **Variable** | **Non-Missing** | **Missing** | **% Missing** |
| --- | --- | --- | --- | --- |
| Outcome | Acute self-limiting respiratory tract infection | 50589 | 0 | 0.00 |
| Registrar | Registrar age | 47661 | 2928 | 5.79 |
|  | Registrar gender | 50577 | 12 | 0.02 |
|  | Registrar full time equivalency | 48293 | 2296 | 4.54 |
|  | Training term/post | 50589 | 0 | 0.00 |
|  | Worked at practice previously | 47969 | 2620 | 5.18 |
|  | Qualified as doctor in Australia | 49848 | 741 | 1.46 |
| Patient | Patient age group | 50034 | 555 | 1.10 |
|  | Patient gender | 49649 | 940 | 1.86 |
|  | Aboriginal and Torres Strait Islander status | 46932 | 3657 | 7.23 |
|  | Non-English speaking background | 46823 | 3766 | 7.44 |
|  | Patient/practice status | 49718 | 871 | 1.72 |
| Consultation | Consultation duration | 47773 | 2816 | 5.57 |
|  | Sought assistance from supervisor | 50589 | 0 | 0.00 |
| Practice | Practice size | 48319 | 2270 | 4.49 |
|  | Practice routinely bulk bills | 48338 | 2251 | 4.45 |
|  | Rurality | 50583 | 6 | 0.01 |
|  | Socio-Economic Indexes for Areas | 50560 | 29 | 0.06 |
|  | Training region | 50589 | 0 | 0.00 |

### Supplementary Table 2. Missing data details from the primary analysis of acute bronchitis diagnoses.

| **Group** | **Variable** | **Non-Missing** | **Missing** | **% Missing** |
| --- | --- | --- | --- | --- |
| Outcome | Acute bronchitis | 5552 | 0 | 0.00 |
| Registrar | Registrar age | 5282 | 270 | 4.86 |
|  | Registrar gender | 5551 | 1 | 0.02 |
|  | Registrar full time equivalency | 5286 | 266 | 4.79 |
|  | Training term/post | 5552 | 0 | 0.00 |
|  | Worked at practice previously | 5271 | 281 | 5.06 |
|  | Qualified as doctor in Australia | 5459 | 93 | 1.68 |
| Patient | Patient age group | 5487 | 65 | 1.17 |
|  | Patient gender | 5442 | 110 | 1.98 |
|  | Aboriginal and Torres Strait Islander status | 5169 | 383 | 6.90 |
|  | Non-English speaking background | 5167 | 385 | 6.93 |
|  | Patient/practice status | 5432 | 120 | 2.16 |
| Consultation | Consultation duration | 5203 | 349 | 6.29 |
|  | Sought assistance from supervisor | 5552 | 0 | 0.00 |
| Practice | Practice size | 5318 | 234 | 4.21 |
|  | Practice routinely bulk bills | 5347 | 205 | 3.69 |
|  | Rurality | 5552 | 0 | 0.00 |
|  | Socio-Economic Indexes for Areas | 5550 | 2 | 0.04 |
|  | Training region | 5552 | 0 | 0.00 |

### Supplementary Table 3. Missing data details from the secondary analysis of acute self-limiting respiratory infection diagnoses.

| **Group** | **Variable** | **Non-Missing** | **Missing** | **% Missing** |
| --- | --- | --- | --- | --- |
| Outcome | Acute self-limiting respiratory tract infection | 43409 | 0 | 0.00 |
| Registrar | Registrar age | 41105 | 2304 | 5.31 |
|  | Registrar gender | 43397 | 12 | 0.03 |
|  | Registrar full time equivalency | 41639 | 1770 | 4.08 |
|  | Training term/post | 43409 | 0 | 0.00 |
|  | Worked at practice previously | 41296 | 2113 | 4.87 |
|  | Qualified as doctor in Australia | 42800 | 609 | 1.40 |
| Patient | Patient age group | 42920 | 489 | 1.13 |
|  | Patient gender | 42594 | 815 | 1.88 |
|  | Aboriginal and Torres Strait Islander status | 40440 | 2969 | 6.84 |
|  | Non-English speaking background | 40386 | 3023 | 6.96 |
|  | Patient/practice status | 42699 | 710 | 1.64 |
| Consultation | Consultation duration | 41001 | 2408 | 5.55 |
|  | Sought assistance from supervisor | 43409 | 0 | 0.00 |
| Practice | Practice size | 41641 | 1768 | 4.07 |
|  | Practice routinely bulk bills | 41623 | 1786 | 4.11 |
|  | Rurality | 43409 | 0 | 0.00 |
|  | Socio-Economic Indexes for Areas | 43409 | 0 | 0.00 |
|  | Training region | 43409 | 0 | 0.00 |

### Supplementary Table 4. Missing data details from the secondary analysis of acute bronchitis diagnoses.

| **Group** | **Variable** | **Non-Missing** | **Missing** | **% Missing** |
| --- | --- | --- | --- | --- |
| Outcome | Acute bronchitis | 4821 | 0 | 0.00 |
| Registrar | Registrar age | 4611 | 210 | 4.36 |
|  | Registrar gender | 4820 | 1 | 0.02 |
|  | Registrar full time equivalency | 4616 | 205 | 4.25 |
|  | Training term/post | 4821 | 0 | 0.00 |
|  | Worked at practice previously | 4601 | 220 | 4.56 |
|  | Qualified as doctor in Australia | 4738 | 83 | 1.72 |
| Patient | Patient age group | 4765 | 56 | 1.16 |
|  | Patient gender | 4731 | 90 | 1.87 |
|  | Aboriginal and Torres Strait Islander status | 4501 | 320 | 6.64 |
|  | Non-English speaking background | 4500 | 321 | 6.66 |
|  | Patient/practice status | 4719 | 102 | 2.12 |
| Consultation | Consultation duration | 4517 | 304 | 6.31 |
|  | Sought assistance from supervisor | 4821 | 0 | 0.00 |
| Practice | Practice size | 4639 | 182 | 3.78 |
|  | Practice routinely bulk bills | 4664 | 157 | 3.26 |
|  | Rurality | 4821 | 0 | 0.00 |
|  | Socio-Economic Indexes for Areas | 4821 | 0 | 0.00 |
|  | Training region | 4821 | 0 | 0.00 |

### Supplementary Table 5: Missingness for models of A) all acute respiratory tract infection and B) acute bronchitis: main analyses and sensitivity analyses.

|  | **Main** | | **Sensitivity** | |
| --- | --- | --- | --- | --- |
| **Model** | **A** | **B** | **A** | **B** |
| Model 1 | 50589 | 5552 | 43409 | 4821 |
| Model 2 | 50589 | 5552 | 43409 | 4821 |
| Model 3 | 45740 | 5005 | 39416 | 4367 |
| Model 4 | 41080 | 4526 | 35552 | 3964 |
| Model 5 | 39021 | 4274 | 33753 | 3740 |
| Model 6 | 38273 | 4206 | 33086 | 3677 |

### Supplementary Table 6. Multivariable regressions for antibiotics prescribed for new acute self-limiting respiratory infection diagnoses (primary)

|  | | | **Model 3** | | **Model 4** | | **Model 5** | | **Model 6** | |
| --- | --- | --- | --- | --- | --- | --- | --- | --- | --- | --- |
| **Variable group** | **Variable** | **Class** | **OR (95% CI)** | **p-value** | **OR (95% CI)** | **p-value** | **OR (95% CI)** | **p-value** | **OR (95% CI)** | **p-value** |
| Registrar variables | Qualified as doctor in Australia | Yes | 0.68 (0.62, 0.75) | <.0001 | 0.68 (0.61, 0.75) | <.0001 | 0.69 (0.62, 0.77) | <.0001 | 0.71 (0.64, 0.80) | <.0001 |
|  | Registrar full time equivalency | Part-time | 0.95 (0.89, 1.02) | 0.1326 | 0.94 (0.88, 1.01) | 0.1190 | 0.94 (0.87, 1.01) | 0.0815 | 0.96 (0.89, 1.03) | 0.2632 |
|  | Registrar age |  | 1.00 (1.00, 1.01) | 0.4785 | 1.00 (0.99, 1.01) | 0.9491 | 1.00 (0.99, 1.01) | 0.7862 | 1.00 (0.99, 1.00) | 0.5353 |
|  | Registrar gender | Female | 1.10 (1.03, 1.18) | 0.0077 | 1.10 (1.02, 1.19) | 0.0119 | 1.09 (1.01, 1.17) | 0.0322 | 1.08 (1.00, 1.16) | 0.0483 |
|  | Training term/post | Term 2 | 1.13 (1.07, 1.20) | <.0001 | 1.14 (1.07, 1.21) | <.0001 | 1.20 (1.12, 1.28) | <.0001 | 1.20 (1.12, 1.28) | <.0001 |
|  | Referent: Term 1 | Term 3 | 0.99 (0.93, 1.06) | 0.8720 | 1.02 (0.95, 1.08) | 0.6562 | 1.07 (1.00, 1.15) | 0.0436 | 1.07 (1.00, 1.15) | 0.0590 |
|  | Worked at practice previously | Yes | 0.99 (0.93, 1.06) | 0.8595 | 1.00 (0.93, 1.07) | 0.8930 | 1.00 (0.93, 1.08) | 0.9721 | 0.97 (0.90, 1.05) | 0.5090 |
| Patient variables | Aboriginal and Torres Strait Islander status | Yes |  |  | 1.40 (1.18, 1.66) | <.0001 | 1.36 (1.14, 1.62) | 0.0005 | 1.31 (1.09, 1.56) | 0.0030 |
|  | Non-English speaking background | Yes |  |  | 0.81 (0.74, 0.89) | <.0001 | 0.81 (0.74, 0.89) | <.0001 | 0.86 (0.78, 0.94) | 0.0014 |
|  | Patient gender | Female |  |  | 1.03 (0.98, 1.07) | 0.2637 | 1.02 (0.97, 1.07) | 0.3866 | 1.01 (0.97, 1.06) | 0.5453 |
|  | Patient age group | 05-14 years |  |  | 1.42 (1.32, 1.54) | <.0001 | 1.48 (1.37, 1.60) | <.0001 | 1.48 (1.37, 1.61) | <.0001 |
|  | Referent: 0-04 years | 15-24 years |  |  | 1.45 (1.34, 1.57) | <.0001 | 1.50 (1.38, 1.62) | <.0001 | 1.51 (1.39, 1.63) | <.0001 |
|  |  | 25-44 years |  |  | 1.53 (1.43, 1.64) | <.0001 | 1.59 (1.49, 1.70) | <.0001 | 1.61 (1.50, 1.73) | <.0001 |
|  |  | 45-64 years |  |  | 1.77 (1.64, 1.90) | <.0001 | 1.78 (1.65, 1.92) | <.0001 | 1.79 (1.66, 1.93) | <.0001 |
|  |  | 65 years+ |  |  | 2.88 (2.62, 3.17) | <.0001 | 2.83 (2.57, 3.12) | <.0001 | 2.82 (2.56, 3.12) | <.0001 |
|  | Patient/practice status | New to practice |  |  | 1.09 (1.00, 1.19) | 0.0500 | 1.07 (0.98, 1.18) | 0.1165 | 1.07 (0.98, 1.18) | 0.1240 |
|  | Referent: Existing patient | New to registrar |  |  | 0.99 (0.94, 1.05) | 0.8299 | 1.01 (0.95, 1.07) | 0.7543 | 1.00 (0.95, 1.06) | 0.9440 |
| Consult variables | Consultation duration |  |  |  |  |  | 1.02 (1.02, 1.02) | <.0001 | 1.02 (1.02, 1.02) | <.0001 |
|  | Sought assistance | Yes |  |  |  |  | 2.09 (1.80, 2.43) | <.0001 | 2.05 (1.77, 2.39) | <.0001 |
| Practice variables | Practice routinely bulk bills | Yes |  |  |  |  |  |  | 0.90 (0.83, 0.98) | 0.0165 |
|  | Practice size | Small |  |  |  |  |  |  | 1.00 (0.93, 1.07) | 0.9439 |
|  | Training region | Region 2 |  |  |  |  |  |  | 0.96 (0.78, 1.17) | 0.6586 |
|  | Referent: Region 1 | Region 3 |  |  |  |  |  |  | 0.76 (0.63, 0.92) | 0.0054 |
|  |  | Region 4 |  |  |  |  |  |  | 0.77 (0.67, 0.88) | 0.0001 |
|  |  | Region 5 |  |  |  |  |  |  | 1.01 (0.73, 1.40) | 0.9526 |
|  |  | Region 6 |  |  |  |  |  |  | 0.70 (0.60, 0.82) | <.0001 |
|  |  | Region 7 |  |  |  |  |  |  | 0.63 (0.54, 0.74) | <.0001 |
|  | Rurality | Inner regional |  |  |  |  |  |  | 1.11 (0.98, 1.26) | 0.0904 |
|  | Referent: Major city | Outer regional remote |  |  |  |  |  |  | 1.33 (1.11, 1.58) | 0.0016 |
|  | Socio-Economic Indexes for Areas |  |  |  |  |  |  |  | 1.01 (1.00, 1.03) | 0.0768 |

### Supplementary Table 7. Multivariable regressions for antibiotics prescribed for new bronchitis diagnosis (primary)

|  | | | **Model 3** | | **Model 4** | | **Model 5** | | **Model 6** | |
| --- | --- | --- | --- | --- | --- | --- | --- | --- | --- | --- |
| **Variable group** | **Variable** | **Class** | **OR (95% CI)** | **p-value** | **OR (95% CI)** | **p-value** | **OR (95% CI)** | **p-value** | **OR (95% CI)** | **p-value** |
| Registrar variables | Qualified as doctor in Australia | Yes | 0.88 (0.69, 1.13) | 0.3047 | 0.86 (0.65, 1.14) | 0.2949 | 0.91 (0.68, 1.21) | 0.5016 | 0.91 (0.67, 1.23) | 0.5376 |
|  | Registrar full time equivalency | Part-time | 0.96 (0.79, 1.18) | 0.7189 | 0.94 (0.75, 1.19) | 0.6117 | 0.98 (0.77, 1.25) | 0.8922 | 0.98 (0.77, 1.26) | 0.8974 |
|  | Registrar age |  | 1.01 (1.00, 1.03) | 0.1584 | 1.01 (0.99, 1.03) | 0.3026 | 1.01 (0.99, 1.03) | 0.2785 | 1.01 (1.00, 1.03) | 0.1345 |
|  | Registrar gender | Female | 1.12 (0.93, 1.34) | 0.2357 | 1.22 (1.00, 1.50) | 0.0541 | 1.28 (1.04, 1.58) | 0.0223 | 1.29 (1.04, 1.60) | 0.0183 |
|  | Training term/post | Term 2 | 1.20 (0.98, 1.47) | 0.0746 | 1.22 (0.97, 1.54) | 0.0827 | 1.15 (0.91, 1.46) | 0.2419 | 1.12 (0.88, 1.43) | 0.3587 |
|  | Referent: Term 1 | Term 3 | 1.07 (0.87, 1.31) | 0.5216 | 1.05 (0.83, 1.33) | 0.6579 | 0.95 (0.75, 1.22) | 0.7077 | 0.94 (0.74, 1.21) | 0.6496 |
|  | Worked at practice previously | Yes | 1.16 (0.94, 1.44) | 0.1700 | 1.17 (0.92, 1.50) | 0.2051 | 1.17 (0.91, 1.51) | 0.2176 | 1.25 (0.95, 1.63) | 0.1049 |
| Patient variables | Aboriginal and Torres Strait Islander status | Yes |  |  | 0.91 (0.51, 1.60) | 0.7350 | 0.91 (0.50, 1.63) | 0.7410 | 1.01 (0.55, 1.84) | 0.9773 |
|  | Non-English speaking background | Yes |  |  | 1.08 (0.76, 1.53) | 0.6652 | 1.08 (0.75, 1.55) | 0.6900 | 1.04 (0.71, 1.51) | 0.8418 |
|  | Patient gender | Female |  |  | 0.93 (0.78, 1.10) | 0.3923 | 0.90 (0.75, 1.07) | 0.2310 | 0.89 (0.75, 1.07) | 0.2166 |
|  | Patient age group | 05-14 years |  |  | 8.77 (5.97, 12.9) | <.0001 | 8.69 (5.83, 12.9) | <.0001 | 8.88 (5.94, 13.3) | <.0001 |
|  | Referent: 0-04 years | 15-24 years |  |  | 9.12 (6.30, 13.2) | <.0001 | 8.24 (5.63, 12.1) | <.0001 | 8.42 (5.71, 12.4) | <.0001 |
|  |  | 25-44 years |  |  | 8.66 (6.60, 11.4) | <.0001 | 8.69 (6.54, 11.6) | <.0001 | 8.94 (6.70, 11.9) | <.0001 |
|  |  | 45-64 years |  |  | 9.83 (7.50, 12.9) | <.0001 | 9.98 (7.53, 13.2) | <.0001 | 10.1 (7.60, 13.4) | <.0001 |
|  |  | 65 years+ |  |  | 11.7 (8.72, 15.7) | <.0001 | 12.1 (8.95, 16.5) | <.0001 | 12.5 (9.14, 17.0) | <.0001 |
|  | Patient/practice status | New to practice |  |  | 1.23 (0.89, 1.72) | 0.2099 | 1.31 (0.93, 1.85) | 0.1183 | 1.30 (0.92, 1.84) | 0.1333 |
|  | Referent: Existing Patient | New to registrar |  |  | 1.19 (0.98, 1.44) | 0.0853 | 1.23 (1.01, 1.50) | 0.0413 | 1.21 (0.98, 1.48) | 0.0701 |
| Consult variables | Consultation duration |  |  |  |  |  | 0.98 (0.97, 0.99) | 0.0001 | 0.98 (0.97, 0.99) | 0.0008 |
|  | Sought assistance | Yes |  |  |  |  | 1.04 (0.70, 1.55) | 0.8402 | 1.03 (0.70, 1.54) | 0.8696 |
| Practice variables | Practice routinely bulk bills | Yes |  |  |  |  |  |  | 1.11 (0.85, 1.44) | 0.4568 |
|  | Practice size | Small |  |  |  |  |  |  | 0.80 (0.65, 0.99) | 0.0424 |
|  | Training region | Region 2 |  |  |  |  |  |  | 0.88 (0.53, 1.47) | 0.6298 |
|  | Referent: Region 1 | Region 3 |  |  |  |  |  |  | 0.71 (0.45, 1.12) | 0.1382 |
|  |  | Region 4 |  |  |  |  |  |  | 1.46 (1.05, 2.03) | 0.0229 |
|  |  | Region 5 |  |  |  |  |  |  | 1.55 (0.66, 3.62) | 0.3161 |
|  |  | Region 6 |  |  |  |  |  |  | 1.08 (0.72, 1.62) | 0.7141 |
|  |  | Region 7 |  |  |  |  |  |  | 0.74 (0.49, 1.12) | 0.1579 |
|  | Rurality | Inner regional |  |  |  |  |  |  | 1.19 (0.87, 1.64) | 0.2772 |
|  | Referent: Major city | Outer regional remote |  |  |  |  |  |  | 1.20 (0.75, 1.91) | 0.4413 |
|  | Socio-Economic Indexes for Areas |  |  |  |  |  |  |  | 1.00 (0.96, 1.04) | 0.9232 |

### Supplementary Table 8. Multivariable regressions for antibiotics prescribed for new acute self-limiting respiratory infection diagnoses (sensitivity)

|  | | | **Model 3** | | **Model 4** | | **Model 5** | | **Model 6** | |
| --- | --- | --- | --- | --- | --- | --- | --- | --- | --- | --- |
| **Variable group** | **Variable** | **Class** | **OR (95% CI)** | **p-value** | **OR (95% CI)** | **p-value** | **OR (95% CI)** | **p-value** | **OR (95% CI)** | **p-value** |
| Registrar variables | Qualified as doctor in Australia | Yes | 0.69 (0.62, 0.77) | <.0001 | 0.68 (0.61, 0.77) | <.0001 | 0.70 (0.62, 0.79) | <.0001 | 0.74 (0.65, 0.83) | <.0001 |
|  | Registrar full time equivalency | Part-time | 0.94 (0.87, 1.01) | 0.1107 | 0.93 (0.86, 1.00) | 0.0509 | 0.92 (0.85, 0.99) | 0.0367 | 0.93 (0.86, 1.01) | 0.0862 |
|  | Registrar age |  | 1.00 (1.00, 1.01) | 0.4933 | 1.00 (0.99, 1.01) | 0.9770 | 1.00 (0.99, 1.01) | 0.7868 | 1.00 (0.99, 1.00) | 0.5241 |
|  | Registrar gender | Female | 1.11 (1.03, 1.19) | 0.0090 | 1.11 (1.02, 1.20) | 0.0123 | 1.09 (1.01, 1.19) | 0.0291 | 1.09 (1.01, 1.19) | 0.0278 |
|  | Training term/post | Term 2 | 1.18 (1.10, 1.25) | <.0001 | 1.17 (1.09, 1.25) | <.0001 | 1.23 (1.14, 1.32) | <.0001 | 1.23 (1.15, 1.32) | <.0001 |
|  | Referent: Term 1 | Term 3 | 1.04 (0.97, 1.11) | 0.2951 | 1.05 (0.98, 1.13) | 0.1744 | 1.11 (1.03, 1.20) | 0.0071 | 1.11 (1.02, 1.19) | 0.0099 |
|  | Worked at practice previously | Yes | 0.97 (0.91, 1.05) | 0.4580 | 0.98 (0.91, 1.06) | 0.5626 | 0.98 (0.90, 1.06) | 0.5682 | 0.96 (0.88, 1.04) | 0.2700 |
| Patient variables | Aboriginal and Torres Strait Islander status | Yes |  |  | 1.29 (1.07, 1.55) | 0.0077 | 1.23 (1.02, 1.49) | 0.0326 | 1.19 (0.98, 1.45) | 0.0732 |
|  | Non-English speaking background | Yes |  |  | 0.82 (0.74, 0.90) | <.0001 | 0.81 (0.73, 0.90) | <.0001 | 0.86 (0.78, 0.96) | 0.0049 |
|  | Patient gender | Female |  |  | 1.02 (0.97, 1.08) | 0.3450 | 1.02 (0.97, 1.07) | 0.5527 | 1.01 (0.96, 1.07) | 0.6226 |
|  | Patient age group | 05-14 years |  |  | 1.44 (1.33, 1.56) | <.0001 | 1.49 (1.37, 1.62) | <.0001 | 1.49 (1.37, 1.62) | <.0001 |
|  | Referent: 0-04 years | 15-24 years |  |  | 1.46 (1.34, 1.58) | <.0001 | 1.50 (1.37, 1.63) | <.0001 | 1.50 (1.38, 1.64) | <.0001 |
|  |  | 25-44 years |  |  | 1.53 (1.42, 1.64) | <.0001 | 1.58 (1.47, 1.71) | <.0001 | 1.59 (1.48, 1.72) | <.0001 |
|  |  | 45-64 years |  |  | 1.78 (1.65, 1.93) | <.0001 | 1.80 (1.66, 1.95) | <.0001 | 1.80 (1.66, 1.96) | <.0001 |
|  |  | 65 years+ |  |  | 2.85 (2.57, 3.15) | <.0001 | 2.80 (2.52, 3.11) | <.0001 | 2.77 (2.49, 3.08) | <.0001 |
|  | Patient/practice status | New to practice |  |  | 1.05 (0.96, 1.16) | 0.2923 | 1.04 (0.94, 1.15) | 0.4216 | 1.05 (0.95, 1.16) | 0.3369 |
|  | Referent: Existing Patient | New to registrar |  |  | 0.98 (0.92, 1.04) | 0.5278 | 1.00 (0.94, 1.06) | 0.9093 | 0.99 (0.93, 1.06) | 0.8556 |
| Consult variables | Consultation duration |  |  |  |  |  | 1.02 (1.02, 1.02) | <.0001 | 1.02 (1.02, 1.02) | <.0001 |
|  | Sought assistance | Yes |  |  |  |  | 2.10 (1.78, 2.46) | <.0001 | 2.06 (1.75, 2.43) | <.0001 |
| Practice variables | Practice routinely bulk bills | Yes |  |  |  |  |  |  | 0.91 (0.83, 1.00) | 0.0393 |
|  | Practice size | Small |  |  |  |  |  |  | 1.02 (0.95, 1.10) | 0.5207 |
|  | Training region | Region 2 |  |  |  |  |  |  | 0.93 (0.75, 1.16) | 0.5313 |
|  | Referent: Region 1 | Region 3 |  |  |  |  |  |  | 0.75 (0.62, 0.92) | 0.0050 |
|  |  | Region 4 |  |  |  |  |  |  | 0.77 (0.67, 0.88) | 0.0002 |
|  |  | Region 5 |  |  |  |  |  |  | 1.06 (0.57, 1.95) | 0.8566 |
|  |  | Region 6 |  |  |  |  |  |  | 0.67 (0.57, 0.79) | <.0001 |
|  |  | Region 7 |  |  |  |  |  |  | 0.64 (0.54, 0.77) | <.0001 |
|  | Rurality | Inner regional |  |  |  |  |  |  | 1.14 (1.00, 1.30) | 0.0473 |
|  | Referent: Major city | Outer regional remote |  |  |  |  |  |  | 1.36 (1.12, 1.65) | 0.0019 |
|  | Socio-Economic Indexes for Areas |  |  |  |  |  |  |  | 1.01 (1.00, 1.03) | 0.0763 |

Supplementary Table 9. Multivariable regressions for antibiotics prescribed for new bronchitis diagnosis (sensitivity)

|  | | | **Model 3** | | **Model 4** | | **Model 5** | | **Model 6** | |
| --- | --- | --- | --- | --- | --- | --- | --- | --- | --- | --- |
| **Variable group** | **Variable** | **Class** | **OR (95% CI)** | **p-value** | **OR (95% CI)** | **p-value** | **OR (95% CI)** | **p-value** | **OR (95% CI)** | **p-value** |
| Registrar variables | Qualified as doctor in Australia | Yes | 0.92 (0.71, 1.20) | 0.5419 | 0.93 (0.69, 1.26) | 0.6364 | 1.00 (0.73, 1.37) | 0.9867 | 1.01 (0.72, 1.40) | 0.9692 |
|  | Registrar full time equivalency | Part-time | 0.88 (0.71, 1.10) | 0.2680 | 0.84 (0.66, 1.08) | 0.1756 | 0.89 (0.68, 1.15) | 0.3682 | 0.87 (0.67, 1.13) | 0.2995 |
|  | Registrar age |  | 1.01 (0.99, 1.03) | 0.2248 | 1.01 (0.99, 1.03) | 0.3992 | 1.01 (0.99, 1.03) | 0.3236 | 1.01 (0.99, 1.04) | 0.1903 |
|  | Registrar gender | Female | 1.07 (0.88, 1.30) | 0.5048 | 1.16 (0.93, 1.45) | 0.1764 | 1.22 (0.97, 1.53) | 0.0934 | 1.25 (0.99, 1.58) | 0.0602 |
|  | Training term/post | Term 2 | 1.29 (1.04, 1.60) | 0.0208 | 1.30 (1.02, 1.66) | 0.0375 | 1.19 (0.92, 1.54) | 0.1826 | 1.14 (0.88, 1.49) | 0.3191 |
|  | Referent: Term 1 | Term 3 | 1.22 (0.97, 1.54) | 0.0864 | 1.24 (0.96, 1.60) | 0.1008 | 1.12 (0.85, 1.47) | 0.4131 | 1.09 (0.83, 1.43) | 0.5528 |
|  | Worked at practice previously | Yes | 1.07 (0.85, 1.35) | 0.5431 | 1.06 (0.81, 1.38) | 0.6662 | 1.06 (0.80, 1.39) | 0.6894 | 1.20 (0.90, 1.60) | 0.2215 |
| Patient variables | Aboriginal and Torres Strait Islander status | Yes |  |  | 1.15 (0.61, 2.15) | 0.6675 | 1.11 (0.58, 2.14) | 0.7564 | 1.32 (0.67, 2.59) | 0.4194 |
|  | Non-English speaking background | Yes |  |  | 1.09 (0.74, 1.59) | 0.6742 | 1.06 (0.71, 1.58) | 0.7829 | 0.97 (0.64, 1.46) | 0.8708 |
|  | Patient gender | Female |  |  | 0.94 (0.78, 1.13) | 0.4948 | 0.91 (0.75, 1.10) | 0.3326 | 0.89 (0.74, 1.09) | 0.2564 |
|  | Patient age group | 05-14 years |  |  | 8.27 (5.49, 12.4) | <.0001 | 8.28 (5.41, 12.7) | <.0001 | 8.77 (5.70, 13.5) | <.0001 |
|  | Referent: 0-04 years | 15-24 years |  |  | 9.37 (6.32, 13.9) | <.0001 | 8.56 (5.69, 12.9) | <.0001 | 8.86 (5.84, 13.4) | <.0001 |
|  |  | 25-44 years |  |  | 8.90 (6.63, 11.9) | <.0001 | 9.06 (6.64, 12.4) | <.0001 | 9.29 (6.79, 12.7) | <.0001 |
|  |  | 45-64 years |  |  | 9.69 (7.26, 12.9) | <.0001 | 9.94 (7.33, 13.5) | <.0001 | 10.2 (7.48, 13.8) | <.0001 |
|  |  | 65 years+ |  |  | 11.6 (8.44, 15.8) | <.0001 | 12.2 (8.77, 17.0) | <.0001 | 12.6 (9.03, 17.7) | <.0001 |
|  | Patient/practice status | New to practice |  |  | 1.11 (0.77, 1.60) | 0.5690 | 1.15 (0.79, 1.69) | 0.4562 | 1.13 (0.77, 1.65) | 0.5269 |
|  | Referent: Existing Patient | New to registrar |  |  | 1.20 (0.98, 1.48) | 0.0804 | 1.24 (1.00, 1.54) | 0.0526 | 1.23 (0.99, 1.53) | 0.0629 |
| Consult variables | Consultation duration |  |  |  |  |  | 0.97 (0.96, 0.98) | <.0001 | 0.97 (0.96, 0.99) | <.0001 |
|  | Sought assistance | Yes |  |  |  |  | 1.07 (0.70, 1.65) | 0.7502 | 1.05 (0.69, 1.62) | 0.8136 |
| Practice variables | Practice routinely bulk bills | Yes |  |  |  |  |  |  | 1.18 (0.88, 1.58) | 0.2794 |
|  | Practice size | Small |  |  |  |  |  |  | 0.84 (0.66, 1.06) | 0.1357 |
|  | Training region | Region 2 |  |  |  |  |  |  | 0.84 (0.48, 1.46) | 0.5376 |
|  | Referent: Region 1 | Region 3 |  |  |  |  |  |  | 0.69 (0.43, 1.12) | 0.1309 |
|  |  | Region 4 |  |  |  |  |  |  | 1.51 (1.07, 2.13) | 0.0187 |
|  |  | Region 5 |  |  |  |  |  |  | 1.79 (0.27, 11.6) | 0.5438 |
|  |  | Region 6 |  |  |  |  |  |  | 1.23 (0.78, 1.94) | 0.3664 |
|  |  | Region 7 |  |  |  |  |  |  | 0.71 (0.44, 1.14) | 0.1579 |
|  | Rurality | Inner regional |  |  |  |  |  |  | 1.29 (0.91, 1.83) | 0.1454 |
|  | Referent: Major city | Outer regional remote |  |  |  |  |  |  | 1.05 (0.64, 1.75) | 0.8375 |
|  | Socio-Economic Indexes for Areas |  |  |  |  |  |  |  | 0.99 (0.94, 1.03) | 0.5589 |

### Supplementary Table 10. Proportion of variability attributable to practice and to registrar in each of the models: A) all acute respiratory tract infection and B) acute bronchitis: main analyses and sensitivity analyses.

|  | **Main** | | | | | | | | |
| --- | --- | --- | --- | --- | --- | --- | --- | --- | --- |
|  | **A** | | | | **B** | | | | |
| **Model** | **Variance Practice** | **Variance Registrar** | **ICC Registrar** | **ICC Practice** | **Variance Practice** | **Variance Registrar** | **ICC Registrar** | **ICC Practice** |  |
| Model 1 | 0.2814 | . | . | 0.0788 | 0.3790 | . | . | 0.1033 |  |
| Model 2 | 0.1308 | 0.5010 | 0.1277 | 0.0333 | 0.1893 | 0.8587 | 0.1979 | 0.0436 |  |
| Model 3 | 0.1142 | 0.4699 | 0.1213 | 0.0295 | 0.2146 | 0.8155 | 0.1888 | 0.0497 |  |
| Model 4 | 0.1025 | 0.4638 | 0.1203 | 0.0266 | 0.2369 | 0.9503 | 0.2122 | 0.0529 |  |
| Model 5 | 0.1118 | 0.4696 | 0.1213 | 0.0289 | 0.2377 | 0.9552 | 0.2131 | 0.0530 |  |
| Model 6 | 0.0869 | 0.4588 | 0.1196 | 0.0226 | 0.2024 | 0.9410 | 0.2123 | 0.0457 |  |

|  | **Sensitivity** | | | | | | | | |
| --- | --- | --- | --- | --- | --- | --- | --- | --- | --- |
|  | **A** | | | | **B** | | | | |
| **Model** | **Variance Practice** | **Variance Registrar** | **ICC Registrar** | **ICC Practice** | **Variance Practice** | **Variance Registrar** | **ICC Registrar** | **ICC Practice** |  |
| Model 1 | 0.2177 | . | . | 0.0621 | 0.3140 | . | . | 0.0871 |  |
| Model 2 | 0.1283 | 0.4840 | 0.1240 | 0.0329 | 0.1851 | 0.8156 | 0.1901 | 0.0431 |  |
| Model 3 | 0.1089 | 0.4691 | 0.1213 | 0.0282 | 0.2066 | 0.8178 | 0.1895 | 0.0479 |  |
| Model 4 | 0.0940 | 0.4675 | 0.1214 | 0.0244 | 0.2199 | 0.9542 | 0.2137 | 0.0493 |  |
| Model 5 | 0.1029 | 0.4790 | 0.1237 | 0.0266 | 0.2178 | 1.0145 | 0.2243 | 0.0482 |  |
| Model 6 | 0.0768 | 0.4708 | 0.1227 | 0.0200 | 0.1811 | 0.9875 | 0.2215 | 0.0406 |  |
