# Supplementary material for: Interpractice variability in antibiotic prescribing for acute respiratory tract infections: a cross-sectional study of Australian early-career general practitioners
Source: BMJ Open. 2025 Aug 3;15(8):e094811. doi: 10.1136/bmjopen-2024-094811 (PMC12320071; doi:10.1136/bmjopen-2024-094811)
Supplement: Supplementary file 2 [file bmjopen-2024-094811.reviewer_comments.pdf]

PEER REVIEW HISTORY

BMJ Open publishes all reviews undertaken for accepted manuscripts. Reviewers are asked to complete a checklist review form and are provided with free text boxes to elaborate on their assessment. These free text comments are reproduced below.

ARTICLE DETAILS

Title (Provisional)

Inter-practice variability in antibiotic prescribing for acute respiratory tract infections: a cross-sectional study of Australian early-career general practitioners

Authors

Turner, Alexandria; van Driel, Mieke L; Mitchell, Ben; Holliday, Elizabeth; Davis, Josh; Tapley, Amanda; Davey, Andrew; Ralston, Anna; Dizon, Jason; Baillie, Emma; Fielding, Alison; Mulquiney, Katie; Clarke, Lisa; Spike, Neil; Magin, Parker

---

VERSION 1 - REVIEW

**Reviewer**	**1**
**Name**	**Llor, Carl**
**Affiliation**	**University of Southern Denmark, Department of Public Health, General Practice.**
**Date**	**04-Jan-2025**
**COI**	**None**

The paper reads well and is well written. The main goal of the paper is statistical, and a review by a statistician would be commendable. The variability among professionals is well-known, including variability at the national level, at the centre level, and at the practice level. However, the study provides numerical values, i.e. intraclass correlation coefficients and mean odds ratios.

I have only two minor comments:

First, the choice of acute bronchitis as a single condition is not clear. It appears that acute bronchitis is the condition where doctors most often deviate from clinical guidelines, but this should be stated more clearly. I also have concerns about the process of diagnosing this condition, as the need for labeling a process is often blurred. Distinguishing a common cold from acute bronchitis can sometimes be challenging. The first aspect of variability is the differing percentage of acute bronchitis cases diagnosed by the doctors themselves. Some doctors diagnose this condition much more frequently than others, and this should be stated more clearly.

What is the generalisability of these results? After reading the attached protocol, I am still unsure how the sample of registrars was selected. The information about this is unclear. It would be helpful to provide more clarity in the methods section. Could you confirm that these data represent the entirety of registrars in Australia? Do you consider that the results obtained could have been different in other countries with different healthcare systems? This should also be acknowledged in the limitations.

**Reviewer**	**2**
**Name**	**Mueller, Yolanda**
**Affiliation**	**University of Lausanne, Department of Family Medicine**
**Date**	**30-Apr-2025**
**COI**	**None**

Specific comments refer to the page number of the pdf (P) and line number.

Methods

P11, l135 The study design is referred to as a “cross-sectional analysis”, which refers to the type of analysis but not the design per se. Design of the ReCEnT study could be specified briefly.

P12, l167: The outcome described is not entirely correct, as the denominator is lacking: is it the number of prescriptions? Prescription frequency (proportion of certain condition where an antibiotic is prescribed)? Number of prescriptions of a systemic antibiotic per clinical encounters coded as …

P12, l170: What is exactly meant by “linked” between ATC code and ICPC-2 code? Is the registrar himself giving the ICPC2 code for each encounter?

P12, l174: why is time not included as a variable? It is likely that prescription frequency has changed over the 10-year period of the study. This is probably an important parameter.

P13, l180: how were the training region coded, considering that they changed in 2016?

P13, l189: this was said previously.

P13, l191: it is not entirely correct to say that analyses were performed at the “level” of problem/diagnosis. Besides, explanations of the spectrum of ICPC2-codes used in the study are important but do not belong to the statistical analysis section per se. The authors should explain how they selected the clinical encounters (I guess based on the ICPC-2 codes), which defines the study population per se.

Also, for those not familiar with ICPC coding, it would be better to mention each code directly after the text, for example “acute upper respiratory infection; R74”, etc. Please do not abbreviate (strep throat) and check spelling (tonsilitis). How do you ensure that these

conditions were new and self-limiting? Did you consider any syndromic ICPC-code (for example RS12 throat symptoms, RS07cough)?

Overall, the statistical analyses could be described more clearly, shortly, and with better structure. The overall methods would probably be clearer if the STROBE checklist was followed in order. There is a constant switch from one section to the other. For example p11, l215: outcomes, then l218 study populations followed by sensitivity analyses, further l223 descriptive statistics.

P14, l211: to me this is a prescription frequency, not a rate. Besides, it is not possible to see a diagnosis, but a patient.

P15, l225: to me, you reported proportions of antibiotic prescriptions. It is interesting to report the distribution, but you should choose between mean and median.

P15, l228: you correctly used a logistic regression model for your binary outcome. Please do not talk about rates. The Laplace approximation is usually used in Bayesian statistics and nonlinear models, and not standard to estimate parameters from a mixed logistic regression model, to my knowledge. Please explain. For which parameter was this used?

P15, l233: complete case analysis does not handle missing data, it just excludes it. As you have many variables in your different models, you should add the number of observations in each model. Differences between models may be due to the exclusion of an increasing number of observations in the more complex models.

P16 l253: I am not personally familiar with the use of median odd ratios and prefer ICCs, but have no objection against its use.

As your focus in on the ICC, it would be useful to understand the respective part of the variance attributable to practice and to registrar. This is not obvious from table 3.

Also, as there is a certain hierarchy between practice factors, registrar and patient factors, how was this handled in the analysis?

P17, l277 Did the registrars consent to the use of the data they collected and reported? Considering they collected clinical data on patient, there are some unanswered ethical issues here.

Results

P18, l283: How was the response rate calculated?

P18, l285: if registrars contributed more than once in the study, how was this handled? For age recorded at first collection period?

P18, l296: consider moving the sensitivity analysis to the supplementary material.

P19. L301: I am not sure I understand what is meant by the estimation of a “mean prescribing rate”: are you sure this is calculated correctly?

P20, l332: it may reflecting small practices, but also the fact that in some practices GPs systematically prescribe (or do not prescribe) for bronchitis. Please comment.

P19, l334: what would you have considered as negligible? How does this compare with the literature?

P21, l362: regarding the comment on number of bronchitis per practice, have you explored the number of pneumonia or bronchitis/pneumoniua ratio? It is not always easy to distinguish between lower respiratory tract infections, and some practices may have "overdiagnosed" pneumonia instead of bronchitis.

I suggest to add a comment on not exploring or adjusting for temporal trends. Also, some health system factors may influence the probability of prescribing an antibiotic. For example, if there is a widely used phone consultation system in a certain region, patients seen by a GP may be more severe compared to a GP who sees everyone. Are these factors homogenous across Australia?

P22, l374: it is not necessary to specify details of the mdoels.

Tables and figures

P30, Table 1: please use capital N for denominator (N=3175, respectively N=2059). Please explain abbreviations in footnote. Please harmonize the number of decimals.

Regarding the year of graduation, it would be interesting to know the number of years since graduation and inclusion in the study.

P34, Table 3: a model is not empty here.

P36, figure 1. I am surprised at the high number of zeroes, but also of 1s for bronchitis. In this case, is a logistic regression still the best model considering the "ceiling" effect?

P42 Supplementary table 5: the different variables that impact prescription, beyond the question of the ICC, are also of interest and could be commented. Consider moving this table to the main results. I am surprised that adding explanatory factors only marginally reduces the ICC.

VERSION 1 - AUTHOR RESPONSE

Reviewer: 1

Comments to the Author:
The paper reads well and is well written. The main goal of the paper is statistical, and a review by a statistician would be commendable. The variability among professionals is well-known, including variability at the national level, at the centre level, and at the practice level. However, the study provides numerical values, i.e. intraclass correlation coefficients and mean odds ratios.

I have only two minor comments:
First, the choice of acute bronchitis as a single condition is not clear. It appears that acute bronchitis is the condition where doctors most often deviate from clinical guidelines, but this should be stated more clearly.

Acute bronchitis is an exemplar acute, self-limiting, respiratory infection for which Australian evidence-based guidelines stipulate no antibiotic prescribing is appropriate.[1] However, results from previous studies in Australian registrars, and the wider GP population, do indeed indicate a high prevalence of antibiotic prescribing for acute bronchitis.[2, 3].

We have added the sentence 'Acute bronchitis was selected as the exemplar acute, self-limiting, respiratory infections for which antibiotics are not indicated.' to lines 132-134 of our introduction. See also, further rationale for the selection of bronchitis in lines 218-222 of our methods section, following on from our selected problems/diagnoses.

I also have concerns about the process of diagnosing this condition, as the need for labeling a process is often blurred. Distinguishing a common cold from acute bronchitis can sometimes be challenging. The first aspect of variability is the differing percentage of acute bronchitis cases diagnosed by the doctors themselves. Some doctors diagnose this condition much more frequently than others, and this should be stated more clearly.

Distinguishing acute bronchitis from common cold can sometimes be a matter of judgement and, as we discuss in response to Reviewer 2, distinguishing acute bronchitis from pneumonia can certainly be challenging, even in Australian general practice which, for most locations, has ready access to CXRs. We have no audit of the accuracy of registrars' diagnoses. But registrars' diagnostic acumen is not the research question our analyses are addressing in this report. We are interested in registrars' prescribing behaviour in response to their having made a diagnosis of acute bronchitis. It is this therapeutic decision-making that we are interested in, particularly when the authoritative Australian guidelines indicate that antibiotics should not be prescribed for acute bronchitis.

We have added 'following their diagnoses of' to the aims section of our introduction (line 131).

What is the generalisability of these results? After reading the attached protocol, I am still unsure how the sample of registrars was selected. The information about this is unclear. It would be helpful to provide more clarity in the methods section. Could you confirm that these data represent the entirety of registrars in Australia? Do you consider that the results obtained could have been different in other countries with different healthcare systems? This should also be acknowledged in the limitations.

The sample frame consisted of all registrars in participating geographically-defined regional training organisations (during 2016-2022, for which we have data, approximately 43-44% of Australian GP registrars).

It was a requirement of registrar training in these regional training organizations that all of these registrars complete ReCEnT for educational purposes. A very small number of registrars did not complete it, having exemption due to illness or other extenuating circumstances) and were excluded from the denominator in our response rate calculations. A further small number did not satisfactorily complete data collection and so did not provide data for these analyses. A further small number of registrars did not provide consent for this data (which was collected primarily for educational use) to also be used for research purposes. Their data was excluded from the analyses presented here (see lines 173-174). For additional clarity, we had added the sentence 'These analyses only include data from registrars who provided consent to use their data for research purposes.' To lines 174-175 of our methods.

Major changes in the structure of GP Training in 2015/2016 affected the geographic footprint of ReCEnT. But since 2016, approximately 43-44% of all Australian registrars have trained in ReCEnT-participating regions. See lines 150-153 under the 'ReCEnT' section of our methods.

The generalizability of these findings to other registrars in other Australian training regions is strong. The participating registrars work towards the same curricula and summative licensure examinations, and the demographics of registrars in the participating training regions were very similar to that of Australian registrars in general– similar in gender, age, rurality/urbanicity, whether Australian or overseas graduate etc.[4] We have added information to this effect (Lines 167-168)

The generalizability to GP vocational training programs in countries other than Australia is uncertain. The sentence 'In terms of generalisability, the results obtained are strongly generalizable to Australian general practice training but may not be so to training programs in other countries, especially those countries that (unlike Australia, the United Kingdom, Ireland, New Zealand, and a number of other countries) do not have apprenticeship-like training models or that have different prescribing regulatory regimens' has been added to the strengths and limitations section of our discussion (line 390-394).

.

Reviewer: 2

Comments to the Author:
Specific comments refer to the page number of the pdf (P) and line number.
Methods
P11, l135 The study design is referred to as a "cross-sectional analysis", which refers to the type of analysis but not the design per se. Design of the ReCEnT study could be specified briefly.

Lines 137-142 of our original manuscript state that this was a cross sectional analysis from the ReCEnT study and that ReCEnT is an ongoing cohort study of GP registrars.

In our revised manuscript, we have now stated 'This was a cross-sectional analysis of data from the Registrar Clinical Encounters in Training (ReCEnT) ***cohort*** *study' (line 138). We have further specified that recent is an i****nception*** *cohort study: 'ReCEnT is an ongoing i****nceptio****n cohort study of GP registrars' (line 142).*

P12, l167: The outcome described is not entirely correct, as the denominator is lacking: is it the number of prescriptions? Prescription frequency (proportion of certain condition where an antibiotic is prescribed)? Number of prescriptions of a systemic antibiotic per clinical encounters coded as …

Our analyses were at the level of problem/diagnosis and the outcome was a dichotomous outcome: was an antibiotic prescribed for this particular instance of the problem/diagnosis? Yes or no. The outcome for our inferential analyses was not number of prescriptions, or frequency of prescription. We did provide descriptive statistics for the proportion of problems/diagnoses for which antibiotics were prescribed.

For clarity, we have added in lines 178-180: 'The outcome of interest was binary: prescription of a systemic antibiotic (defined as J01 in the Anatomical Therapeutic Chemical (ATC) classification system)[5] versus a systemic antibiotic not being prescribed.'

P12, l170: What is exactly meant by "linked" between ATC code and ICPC-2 code? Is the registrar himself giving the ICPC2 code for each encounter?

The registrars do not assign ICPC-2 codes for each problem/diagnosis. ICPC-2PLUS is quite a complex coding system and registrars record data contemporaneously immediately after consultations (that is, during their working day). The registrars are instructed to describe the problem/diagnosis in their own words as accurately as they can. The registrars' entries for the problems/diagnoses they manage are then coded to the ICPC-2PLUS system by a team of trained data coders.[6, 7] The registrars' entries for medicines prescribed are similarly coded by our data coders to the ATC system. We have added information regarding this to our text (lines 161-162): 'Registrars describe each problem/diagnosis in their own words as accurately as possible. Registrars' responses (for problems/diagnoses) are coded to the International Classification of Primary Care-2 PLUS (ICPC-2PLUS) system by trained coders.

When recording medicines prescribed, registrars record for which problem/s the medicine was prescribed. That is, medicines prescribed are linked in the registrar's contemporaneous recording to the problems/diagnoses for which they were prescribed.

For clarity, changes outlining these processes have been added to lines 160 and 183-185 of the revised manuscript: '…any prescribing performed (specifically linked by registrars in their data recording to the relevant problems/diagnoses that prompted the prescription).'

We have also edited lines 205 and 210 to read 'ICPC-2PLUS' for consistency.

P12, l174: why is time not included as a variable? It is likely that prescription frequency has changed over the 10-year period of the study. This is probably an important parameter.

Year of consultation was specified a priori as an explanatory to be adjusted for in the analysis. However, its inclusion led to convergence issues in the models, preventing estimation of standard errors for the registrar and practice-level random effects. When year was removed, these standard errors could be successfully estimated. As valid estimation of these random

effects is essential for calculating MORs and ICCs, the year variable was ultimately excluded from the final models.

We agree that this is a potential limitation, but anticipate time trends would not have a marked influence on measures of inter-practice variability (which, in themselves, are unlikely to exhibit substantial time trends). We have now noted this in our 'limitations' section in lines 386-387: 'Our analyses cover a 10-year period. It is possible there were changes in prescribing habits over time that varied between practices; however, we anticipate such changes would have negligible impact on measures of inter-practice variability.'

P13, l180: how were the training region coded, considering that they changed in 2016?

Some regions were consistent throughout the data collection period. There was discontinuity with some other regions. We have adjusted for this by including the 'region' variable in our analyses.

P13, l189: this was said previously.

The sentence 'ReCEnT data from 2010 to 2020 were included in these analyses' has now been removed from the statistical analysis section.

P13, l191: it is not entirely correct to say that analyses were performed at the "level" of problem/diagnosis. Besides, explanations of the spectrum of ICPC2-codes used in the study are important but do not belong to the statistical analysis section per se. The authors should explain how they selected the clinical encounters (I guess based on the ICPC-2 codes), which defines the study population per se.

For the ReCEnT project, the study population comprises registrars, with consultations and problems/diagnoses nested within registrars. In these multilevel data, we use the term "level" to refer to the conventional "unit of inference", or the level at which we are making conclusions about our data. Analyses of ReCEnT data are frequently performed either at the consultation level, or at the problem/diagnosis level. For these analyses, the analysis population was all ARTI problems/diagnoses, or all bronchitis problems/diagnoses, respectively. For each individual problem/diagnosis, the binary outcome factor was management with an antibiotic prescription (yes/no).

The analysis population for the analyses we have presented here is problems/diagnoses defined by the ICPC-2PLUS codes as we have presented them. Thus, we do believe that the analysis section is the appropriate place to have this statement of which codes defined the analysis population.

Also, for those not familiar with ICPC coding, it would be better to mention each code

directly after the text, for example "acute upper respiratory infection; R74", etc. Please do not abbreviate (strep throat) and check spelling (tonsilitis).

Under our revised 'Analysis population' heading, the explanation of included ICPC-2PLUS codes (lines 204-209) now reads: 'Analyses were performed at the problem/diagnosis level and were restricted to the following definitions and ICPC-2PLUS codes:

- *New, self-limiting aRTIs (acute upper respiratory tract infection; R74, acute bronchitis/bronchiolitis; R78, sinusitis acute/chronic; R75, streptococcal throat;R72, tonsillitis acute; R76, and acute otitis media/myringitis; H71 for one set of analyses (here-after referred to as 'aRTIs')'*

How do you ensure that these conditions were new and self-limiting? Did you consider any syndromic ICPC-code (for example RS12 throat symptoms, RS07cough)?

Registrars specifically record whether the conditions are new or existing.[6, 7] 'New' is defined as the first time the episode has presented to a healthcare provider. These acute respiratory tract infections are, by definition, self-limiting.

We excluded throat symptoms and cough as these are non-specific problem formulations and are very unlikely to have been recorded by registrars for acute respiratory infections.

Overall, the statistical analyses could be described more clearly, shortly, and with better structure. The overall methods would probably be clearer if the STROBE checklist was followed in order. There is a constant switch from one section to the other. For example p11, l215: outcomes, then l218 study populations followed by sensitivity analyses, further l223 descriptive statistics.

We have re-ordered the statistical analyses section of the Methods. The order of content is now as per the 'Statistical Methods' section of STROBE.

We have separately labelled descriptive and inferential statistical methods used. And shifted the sensitivity analyses material to the end of the Statistical Analysis section.

We have moved the statement regarding missing data to later in the section.

We note, though, that STROBE is a checklist of content items for manuscripts, not a guide to how to organize a manuscript: 'The STROBE Statement should not be interpreted as an attempt to prescribe the reporting of observational research in a rigid format'.[8] And we have added an 'Analysis population' subheading, which, as per our previous response, we feel belongs in the statistics section (given the complexities around our study population being registrars, but the study population for this specific analysis being 'problems/diagnoses' as you suggested earlier).

P14, l211: to me this is a prescription frequency, not a rate. Besides, it is not possible to see a diagnosis, but a patient.

We have changed our wording throughout manuscript as appropriate. We agree it is not technically a rate, but we considered use of the word sufficient for readers' understanding, particularly as it is used in this way in other manuscripts [9-11] in notable journals (including BMJ Open).

We also considered it appropriate for reducing word count (i.e. 'antibiotic prescribing rate' should ideally be extended to 'proportion of problems for which antibiotics were prescribed'). However, using the words frequency and percentage is also suitable.

The word 'rate' has been changed to:
- frequency in line 39
- percentages in line 56
- percentages in line 61
- frequencies in line 70
- frequencies in line 100
- percentage in line 102
- frequencies in line 105
- frequencies in line 109
- frequencies in line 124
- frequencies in line 125
- proportions in line 216
- proportion in line 221
- proportions in line 228
- odds in line 265
- percentage in lines 320-321
- percentages in lines 324-326
- percentage in lines 346-349
- odds in line 370
- behaviour in line 386
- percentages in line 426
- percentage in line 439
- proportions in figure 1 and figure 1 caption (lines 647-648)

P15, l225: to me, you reported proportions of antibiotic prescriptions. It is interesting to report the distribution, but you should choose between mean and median.

Yes, we have certainly reported proportions. Depending on the distribution of the variable, mean or median, or both, are appropriate. The two statistics provide different information about the distribution. We have changed our wording under 'descriptive statistics' to read: 'Descriptive statistics were reported as mean and standard deviation (SD) or median and interquartile range (IQR), as appropriate, for continuous variables and as frequencies with proportions for categorical variables.' (lines 225-227).

P15, l228: you correctly used a logistic regression model for your binary outcome. Please do not talk about rates.

As above – wording has been changed throughout manuscript.

The Laplace approximation is usually used in Bayesian statistics and nonlinear models, and not standard to estimate parameters from a mixed logistic regression model, to my knowledge. Please explain. For which parameter was this used?

We would like to clarify that when we refer to the Laplace approximation in the methods, we are referring to the estimation procedure used to obtain parameter estimates for all parts of the mixed logistic regression model. This includes both fixed effects (e.g., patient characteristics such as age and gender) and random effects (i.e., variation in the outcome attributable to registrar and practice).

The Laplace approximation is a widely implemented and accepted method for fitting mixed models, available in many statistical software packages. Its use is not a non-conventional approach, and we believe it is appropriate for our study due to its computational efficiency compared to other estimation procedures. Given our large sample size and the inclusion of two levels of random effects (registrar and practice), this method was chosen to reduce computational burden and facilitate model convergence within reasonable timeframes.

P15, l233: complete case analysis does not handle missing data, it just excludes it. As you have many variables in your different models, you should add the number of observations in each model. Differences between models may be due to the exclusion of an increasing number of observations in the more complex models.

We have added this information regarding missingness of data in each model as Supplementary Table 5 and referred to it in line 341 of our revised text.

P16 l253: I am not personally familiar with the use of median odd ratios and prefer ICCs, but have no objection against its use. As your focus in on the ICC, it would be useful to understand the respective part of the variance attributable to practice and to registrar. This is not obvious from table 3.

With regard to the proportion of variance attributable to practice in the ICC, this is directly observable by considering the ICC presented in table 3 itself. If you recall the formulas presented in the Intra-class correlations section of the manuscript, for model 1:

ICC = $\frac{\sigma_P^2}{\sigma_P^2+3.29}$

And for model 2:

ICC = $\frac{\sigma_P^2}{\sigma_P^2+\sigma_R^2+3.29}$

By this formulation, the ICC represents the proportion of the variation attributable to practice.

We agree with the reviewer that the variance attributable to registrar is not immediately obvious. This can be calculated by substituting variance attributable to registrar into the numerator of the formula above. Note that by model construction, such a calculation is not applicable to model 1.

Please see the tables below:

	**Main**							
	**All aRTIs**				**Acute bronchitis**			
**Model**	**Variance Practice**	**Variance Registrar**	**ICC Registrar**	**ICC Practice**	**Variance Practice**	**Variance Registrar**	**ICC Registrar**	**ICC Practice**
Model 1	0.2814	.	.	0.0788	0.3790	.	.	0.1033
Model 2	0.1308	0.5010	0.1277	0.0333	0.1893	0.8587	0.1979	0.0436
Model 3	0.1142	0.4699	0.1213	0.0295	0.2146	0.8155	0.1888	0.0497
Model 4	0.1025	0.4638	0.1203	0.0266	0.2369	0.9503	0.2122	0.0529
Model 5	0.1118	0.4696	0.1213	0.0289	0.2377	0.9552	0.2131	0.0530
Model 6	0.0869	0.4588	0.1196	0.0226	0.2024	0.9410	0.2123	0.0457

	Sensitivity							
	All aRTIs				Acute bronchitis			
Model	Variance Practice	Variance Registrar	ICC Registrar	ICC Practice	Variance Practice	Variance Registrar	ICC Registrar	ICC Practice
Model 1	0.2177	.	.	0.0621	0.3140	.	.	0.0871
Model 2	0.1283	0.4840	0.1240	0.0329	0.1851	0.8156	0.1901	0.0431
Model 3	0.1089	0.4691	0.1213	0.0282	0.2066	0.8178	0.1895	0.0479
Model 4	0.0940	0.4675	0.1214	0.0244	0.2199	0.9542	0.2137	0.0493
Model 5	0.1029	0.4790	0.1237	0.0266	0.2178	1.0145	0.2243	0.0482
Model 6	0.0768	0.4708	0.1227	0.0200	0.1811	0.9875	0.2215	0.0406

We have added this information as Supplementary Table 10, and referred to it in lines 342-343 of our revised manuscript.

Also, as there is a certain hierarchy between practice factors, registrar and patient factors, how was this handled in the analysis?

We note that in the methods, we state that we used mixed-effects logistic regression models with random effects to account for repeated measures on registrars and practices: See lines

237-238: "These models included random effects to account for repeated measures on registrars and practices over time"

We would like to further clarify that mixed-effects models can alternatively be called hierarchical models or multilevel models. These models explicitly account for the hierarchical nature of the data through the inclusion of the random effects.

P17, l277 Did the registrars consent to the use of the data they collected and reported? Considering they collected clinical data on patient, there are some unanswered ethical issues here.

Lines 173-174 state that 'Registrars are also given the option to provide consent for their data to also be used for research purposes.' A further sentence has been added to lines 174-175 of our revised manuscript: 'These analyses only include data from registrars who provided consent to use their data for research purposes.'

Ethic committees who have overseen the study have determined that the participants in the study are registrars. Patients are not participants and their consent is not required. Our study conduct also meets all patient privacy requirements.

Results

P18, l283: How was the response rate calculated?

The denominator was all Term1,2,3 registrars eligible for participation: that is, all registrars who didn't have an exemption.

The numerator was participants: that is, these registrars minus those who didn't provide data or didn't consent to their data being used for research purposes.

P18, l285: if registrars contributed more than once in the study, how was this handled? For age recorded at first collection period?

Analysis was at the level of problem/diagnosis. Clustering of problems/diagnoses within registrars was adjusted for via mixed-effects logistic regression models with random effects to account for repeated measures on registrars, as above.

Age was calculated for each registrar at the beginning of each 6-monthly data collection period.

P18, l296: consider moving the sensitivity analysis to the supplementary material.

We feel it is important to have both presented in the main text. As mentioned in lines 290-291: 'This sensitivity analysis was performed to evaluate the robustness of results from primary

analysis to reduce potential bias by the inclusion of practices contributing smaller numbers of respiratory infections.'

The number of registrar rounds is a potential source of bias in our findings, as illustrated in Figure 1. It is important for us to have reported in the main text, the sensitivity analysis, to demonstrate that our primary analysis is robust.

P19. L301: I am not sure I understand what is meant by the estimation of a "mean prescribing rate": are you sure this is calculated correctly?

This was mean of the individual practice percentages.

For example, for each practice in the data, the proportion of problems where antibiotics were prescribed for bronchitis was calculated e.g. practice 1 was 20%, practice 2 was 30%. Then the mean was calculated based off all these proportions. In the example above, the mean would be 25% ((20 + 30) / 2).

P20, l332: it may reflecting small practices, but also the fact that in some practices GPs systematically prescribe (or do not prescribe) for bronchitis. Please comment.

Yes, that some practices may systematically prescribe or not prescribe for bronchitis is the basis of our research question. Previous analyses in this data set[3, 12] as well as qualitative research suggests registrars prescribe, against their better judgment, to be consistent with the prescribing practice of their supervisors and other GPs in the practice.[13, 14]

P19, l334: what would you have considered as negligible? How does this compare with the literature?

With ICCs, anything that's conclusively above 0 indicates clustering. It's certainly difficult to define 'non-negligible ICCs', but we note that values above zero (even when quite small) are meaningful in clustered designs where they lead to design effects above 1 and reduce the effective sample size. Thus, in our revised manuscript we have linked the statement re 'non-negligible' correlation with our later comparisons with the literature regarding ICCs in general practice trials: '(see below, 'Interpretation within the context of the wider literature', for a discussion of ranges of ICC values)'. That is, the statement regarding 'non-negligible' ICCs is in the 'Statement of principal findings' section of our manuscript (line 356). The discussion of how these compare with the literature is in the 'Interpretation within the context of the wider literature' section (lines 403-421).

P21, l362: regarding the comment on number of bronchitis per practice, have you explored the number of pneumonia or bronchitis/pneumoniua ratio? It is not always easy to

distinguish between lower respiratory tract infections, and some practices may have "overdiagnosed" pneumonia instead of bronchitis.

We hadn't looked at the ratio of pneumonia to bronchitis. The accuracy of registrars' diagnoses was not our research question (though noting, in Australia, GPs generally have prompt access to X-Ray facilities and suspected outpatient pneumonia can usually be diagnosed radiographically). Authoritative Australian guidelines recommend strongly against prescribing antibiotics for acute bronchitis. For our analyses, we accept the registrar's diagnosis. If registrars diagnosed 'acute bronchitis', our research question is whether they then prescribed antibiotics for that diagnosis.

I suggest to add a comment on not exploring or adjusting for temporal trends. Also, some health system factors may influence the probability of prescribing an antibiotic. For example, if there is a widely used phone consultation system in a certain region, patients seen by a GP may be more severe compared to a GP who sees everyone. Are these factors homogenous across Australia?

Our analyses cover a 10-year period. It is possible that any changes in prescribing habits over that time may not have been consistent across participating practices. We have added 'Additionally, our analyses cover a 10-year period. It is possible that any changes in prescribing habits over that time may not have been consistent across practices.' to lines 386-389.

P22, l374: it is not necessary to specify details of the mdoels.

We have removed model numbers from lines 403-404 and 424-425.

Tables and figures
P30, Table 1: please use capital N for denominator (N=3175, respectively N=2059). Please explain abbreviations in footnote.

We have made this change in Table 1 (line 629).

Please harmonize the number of decimals.

This has been adjusted.

Regarding the year of graduation, it would be interesting to know the number of years since graduation and inclusion in the study.

Only one of these two parameters could be included in the analyses. There is very little difference in these given that terms 1 and 2 and terms 2 and 3 are only separated by 6 months each for full time registrars (the large majority of registrars).

P34, Table 3: a model is not empty here.

Models 1 and 2 are 'empty' models defined as without adjusting for any explanatory variables. We have added to our "model construction" section of the methods (lines 277-280)

P36, figure 1. I am surprised at the high number of zeroes, but also of 1s for bronchitis. In this case, is a logistic regression still the best model considering the "ceiling" effect?

We note that the unit of analysis for this study was diagnosis. If you recall from the results for example, there were 50589 aRTI diagnoses, with 18309 having the event of interest. That is to say that this binary outcome has 18309 in the "1" category and 32280 in the "0" category. These numbers are reasonable for a logistic regression.

Figure 1 are descriptive statistics on the level of practice, a completely different unit of analysis to the models themselves. The "ceiling" effect the reviewer is noticing is the result of aggregating diagnoses to the level of practice. For example, some practices may see very few diagnoses of bronchitis and prescribe/not prescribe antibiotics in every case, which leads to the high amounts of 1's and 0's in the graph.

Since the unit of analysis is completely different between the descriptive statistics and the models, the "ceiling" effect the reviewer is noticing is irrelevant to the modelling. If the models were instead on the level of practice such that we were directly modelling these proportions as the outcome, then the "ceiling" effect the reviewer is noticing would be a relevant and serious consideration.

What these figures do highlight however, is that there is considerable variation between practices in prescribing behaviour. This apparent variation in the practices is a motivating reason why practice is included as a random effect in the models.

P42 Supplementary table 5: the different variables that impact prescription, beyond the question of the ICC, are also of interest and could be commented. Consider moving this table to the main results. I am surprised that adding explanatory factors only marginally reduces the ICC.

We cannot select one out of our four multivariable analyses to present in the main results, unfortunately we also can't present all four multivariable analyses as we are already at BMJ Open's figure and table limit of 5.

Our interpretation of the relative stability of the ICCs and MORs with adjustment for potential confounding is that practice culture is an unmeasured variable. This is mentioned in our implications section and is a major finding in terms of possible actions arising from our analyses – promotion of antibiotic stewardship by registrars requires more than education of registrars: practice-level initiatives and supervisor education will be vital components of a multifaceted approach.

Thank you again for the opportunity to revise and resubmit our manuscript,

Yours sincerely

Alexandria Turner and Parker Magin, for the authors

References

1. Therapeutic Guidelines of Australia. eTG complete. Electronic therapeutic guidelines complete. North Melbourne: Therapeutic Guidelines Ltd.; 2002.
2. Australian Comission on Safety and Quality in Healthcare. AURA 2021- The Fourth Australian Report on Antimicrobial use and Resistance in human health. Sydney: ACSQH, 2021.
3. Baillie EJ, Merlo G, Magin P, Tapley A, Mulquiney KJ, Davis JS, et al. Antibiotic prescribing for upper respiratory tract infections and acute bronchitis: a longitudinal analysis of general practitioner trainees. Fam Pract. 2022 May 28. PMID: 35640041. doi: 10.1093/fampra/cmac052.
4. Taylor R, Clarke, L., Radloff, A. Australian General Practice Training Program: National report on the 2020 National Registrar Survey. In: Health AGDo, editor. 2020.
5. World Health Organisation. Anatomical Therapeutic Chemical (ATC) Classification. Available from: https://www.who.int/tools/atc-ddd-toolkit/atc-classification.
6. Davey A, Tapley A, van Driel M, Holliday E, Fielding A, Ball J, et al. The Registrar Clinical Encounters in Training (ReCEnT) cohort study: updated protocol. BMC Primary Care. 2022 2022/12/16;23(1):328. doi: 10.1186/s12875-022-01920-7.
7. Magin P, Morgan S, Henderson KM, Tapley A, Scott J, Spike NA, et al. The Registrars' Clinical Encounters in Training (ReCEnT) project: educational and research aspects of documenting general practice trainees' clinical experience. Aust Fam Physician. 2015 08/25;44:681-4.
8. von Elm E, Altman DG, Egger M, Pocock SJ, Gøtzsche PC, Vandenbroucke JP, et al. The Strengthening the Reporting of Observational Studies in Epidemiology (STROBE) Statement: Guidelines for Reporting Observational Studies. PLOS Medicine. 2007;4(10):e296. doi: 10.1371/journal.pmed.0040296.
9. McCullough AR, Pollack AJ, Plejdrup Hansen M, Glasziou PP, Looke DF, Britt HC, et al. Antibiotics for acute respiratory infections in general practice: comparison of prescribing rates with guideline recommendations. Med J Aust. 2017 Jul 17;207(2):65-9. PMID: 28701117. doi: 10.5694/mja16.01042.
10. Stuart B, Brotherwood H, Van't Hoff C, Brown A, van den Bruel A, Hay AD, et al. Exploring the appropriateness of antibiotic prescribing for common respiratory tract infections in UK primary care. J Antimicrob Chemother. 2020 Jan 1;75(1):236-42. PMID: 31637421. doi: 10.1093/jac/dkz410.
11. Gulliford MC, Dregan A, Moore MV, Ashworth M, Staa TV, McCann G, et al. Continued high rates of antibiotic prescribing to adults with respiratory tract infection:

survey of 568 UK general practices. BMJ Open. 2014 Oct 27;4(10):e006245. PMID: 25348424. doi: 10.1136/bmjopen-2014-006245.
12. Magin PJ, Morgan S, Tapley A, Henderson KM, Holliday EG, Ball J, et al. Changes in early-career family physicians' antibiotic prescribing for upper respiratory tract infection and acute bronchitis: a multicentre longitudinal study. Fam Pract. 2016 Aug;33(4):360-7. PMID: 27095798. doi: 10.1093/fampra/cmw025.
13. Dallas A, van Driel M, van de Mortel T, Magin P. Antibiotic prescribing for the future: exploring the attitudes of trainees in general practice. Br J Gen Pract. 2014 Sep;64(626):e561-7. PMID: 25179070. doi: 10.3399/bjgp14X681373.
14. Deckx L, Anthierens S, Magin PJ, Morgan S, McArthur L, Yardley L, et al. Focus on early-career GPs: Qualitative evaluation of a multi-faceted educational intervention to improve antibiotic prescribing. Family Practice. 2018;35(1):99-104. doi: 10.1093/fampra/cmx074.

VERSION 2 - REVIEW

Reviewer **1**

Name **Llor, Carl**

Affiliation **University of Southern Denmark, Department of Public Health, General Practice.**

Date **10-Jul-2025**

COI

The queries were properly addressed. I don't have any further questions.
